# The Gyaros island marine reserve: A biodiversity hotspot in the eastern Mediterranean Sea

**DOI:** 10.1371/journal.pone.0262943

**Published:** 2022-02-03

**Authors:** Dimitrios Damalas, Caterina Stamouli, Nikolaos Fotiadis, Maria Kikeri, Vasiliki Kousteni, Danai Mantopoulou-Palouka

**Affiliations:** 1 Hellenic Centre for Marine Research, Institute of Marine Biological Resources and Inland Waters, Heraklion, Greece; 2 Hellenic Centre for Marine Research, Institute of Marine Biological Resources & Inland Waters, Athens, Greece; 3 Fisheries Research Institute, Hellenic Agricultural Organization Demeter, Kavala, Greece; Technological Educational Institute of Western Greece, GREECE

## Abstract

Since July 2019, Gyaros island in the central Aegean Sea, enjoys the status of a partial Marine Protected Area (MPA), allowing for exploitation by small-scale fishers following specific spatio-temporal restrictions. The need for assessing the effectiveness of the MPA in the future, led MAVA Foundation to fund a knowledge survey project aiming to serve as a baseline for future reference. A series of experimental fishing surveys took place with static nets, the outcomes of which are presented herein. From June 2018 to September 2020, a series of 8 fishing excursions with a total of 40 experimental fishing sets with bottom static nets were realized in 5 set locations around Gyaros island, inside the MPA protection zone. A total of 75 species were identified; the most abundant species, in terms of biomass, being: parrotfish*-Sparisoma cretense*, red scorpionfish-*Scorpaena scrofa*, common spiny lobster-*Palinurus elephas*, red porgy-*Pagrus pagrus*, little tunny–-*Euthynnus alletteratus*, Mediterranean moray-*Muraena helena*, lesser spotted dogfish -*Scyliorhinus canicula*, forkbeard-*Phycis phycis*, surmullet-*Mullus surmuletus*, common cuttlefish-*Sepia officinalis* and common Pandora-*Pagellus erythrinus*. A comparison with similar data in adjacent areas outside the MPA allowed for assessing the effectiveness of the MPA based on four indicators: species diversity index, species relative biomass index, key predator species abundance, and alien fish abundance. Based solely on the experimental fishing trials, the MPA seems to be functioning, since both species diversity and abundance were higher within the protected area. However, its performance may still not be considered as optimal, as this is indicated by the large proportion of undersized key predators (e.g. groupers), although more abundant and larger than the ones residing outside the MPA.

## Introduction

Marine Protected Areas (MPAs) are considered one of the easiest management strategies to enforce, since they do not require setting up a maze of regulatory restrictions (gear configuration, vessel capacity restrictions, minimum landing sizes, input and output controls, etc.), which can be confusing both to the fishing industry and the general public [1]. There are more than 11,000 MPAs around the world with a coverage of circa 4% of the marine regions. A total of 1140 MPAs are located in the Mediterranean; only 76 of them enjoy fully protected status and have a quite small average size of just 5 km^2^ [2].

**Fig 1:**
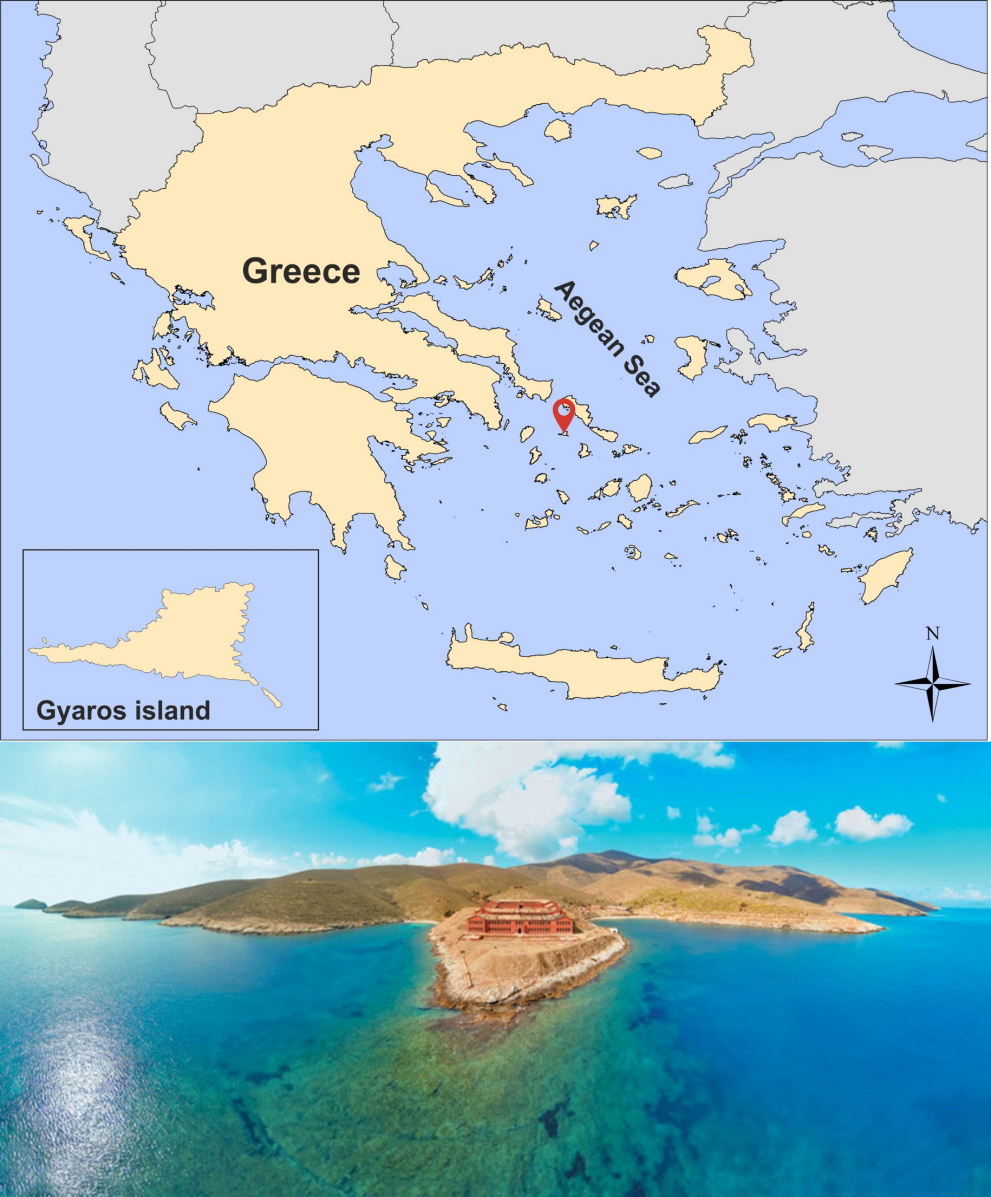
top: Location of Gyaros island (Greece basemap reprinted from Kavadas et al., 2012 - https://doi.org/10.12681/mms.324 under a CC BY license, with permission from Stefanos Kavadas-HCMR, original copyright 2004) bottom: aerial view of the island with the deserted prison complex (source: George Stefanou/WWF Greece).

The marine area surrounding Gyaros island in the central Aegean Sea (eastern Mediterranean Sea) is the most recently established Mediterranean MPA. Gyaros with an area of 17 km^2^ is an arid, deserted island in the northern Cyclades, situated 9 nautical miles from the closest island of Syros (Fig 1). The island has a dark history as it served as a place of exile since the Roman era and during the recent past. After the WWII, Gyaros was established as a concentration camp for displacing political prisoners up until 1974. Afterwards it was converted to a firing range for the Hellenic Navy up to 2002. In 2011, Gyaros and the surrounding marine area of three (3) nautical miles from its coastline, was listed among the European Natura 2000 Network sites and was established as a Wildlife Refuge. Although access to other human activities was limited or restricted and Gyaros should have been enjoying a particular ‘protected’ status for more than five decades [3], a recent study during 2014–2016 [4] identified large number of cases of illegal fishing in the area and moreover the state of fish stocks did not show significant differences with other areas that are normally fished and which are even closer to residential areas or fishing ports [5].

In 2016, a consortium of stakeholders, consisting of 15 members (among them fishers’ associations from surrounding islands, the Ministry of Environment, local authorities, scientific bodies and Non-Governmental Organizations-NGOs) initiated working on a plan to establish Gyaros island marine region as an MPA. The whole endeavour was undertaken by World Wildlife Fund-WWF Greece and was the final aspiration of the CYCLADES LIFE project (http://cycladeslife.gr/). The initiative originated mainly under the recent findings that Gyaros hosts a sizeable Mediterranean monk seal (*Monachus monachus*) breeding colony, apparently the largest in the Mediterranean Sea [6, 7]. During the two-year consultation period, six consortium meetings were realized; it became obvious that the local fishing communities were the most reluctant, expressing openly their scepticism and concerns. Most of these concerns were related to the frequent interaction with Mediterranean monk seals damaging both their nets and catch. In their mindset, providing a sanctuary to monk seals would only lead to larger seal populations and associated income loss. By the end of 2017, a common ground was found among all parties after agreeing on a trade-off between a ‘No-Take-Zone’ and ‘full-access to fishing’. A group visit to the Torre Guaceto MPA in Italy to exchange ideas with local fishers played a key role towards easing Greek fishers’ reservations. Finally, a five-month access to small-scale fishing under specific conditions (zoning system plus a list of conservation measures) was put forward to the national authorities.

Since 2019, following the Ministerial Decree 389/4.7.2019., Gyaros has been declared as an MPA enjoying the status of a partially protected MPA, allowing spatio-temporal access to small-scale fishers where specific exploitation activities are permitted and regulated.

In order to have a baseline of the Gyaros living marine resources for assessing future status and functioning of the MPA, the Hellenic Centre for Marine Research was granted by the MAVA Foundation the project “*Gyaros MPA fisheries knowledge survey*: *assessing a pristine Mediterranean biodiversity hotspot*” (Grant Agreement 17114). MAVA was created in 1994 as a key funder of global conservation. The project lasted for three years and included experimental fishing surveys, ichthyoplankton surveys, hydroacoustic surveys, underwater visual census surveys and public outreach activities with involved stakeholders.

The outcomes of a series of experimental fishing surveys, during 2018–2020, in the Gyaros MPA and a comparison with the surrounding areas outside the MPA are presented herein.

## Materials and methods

### Sampling inside the MPA

**Fig 2:**
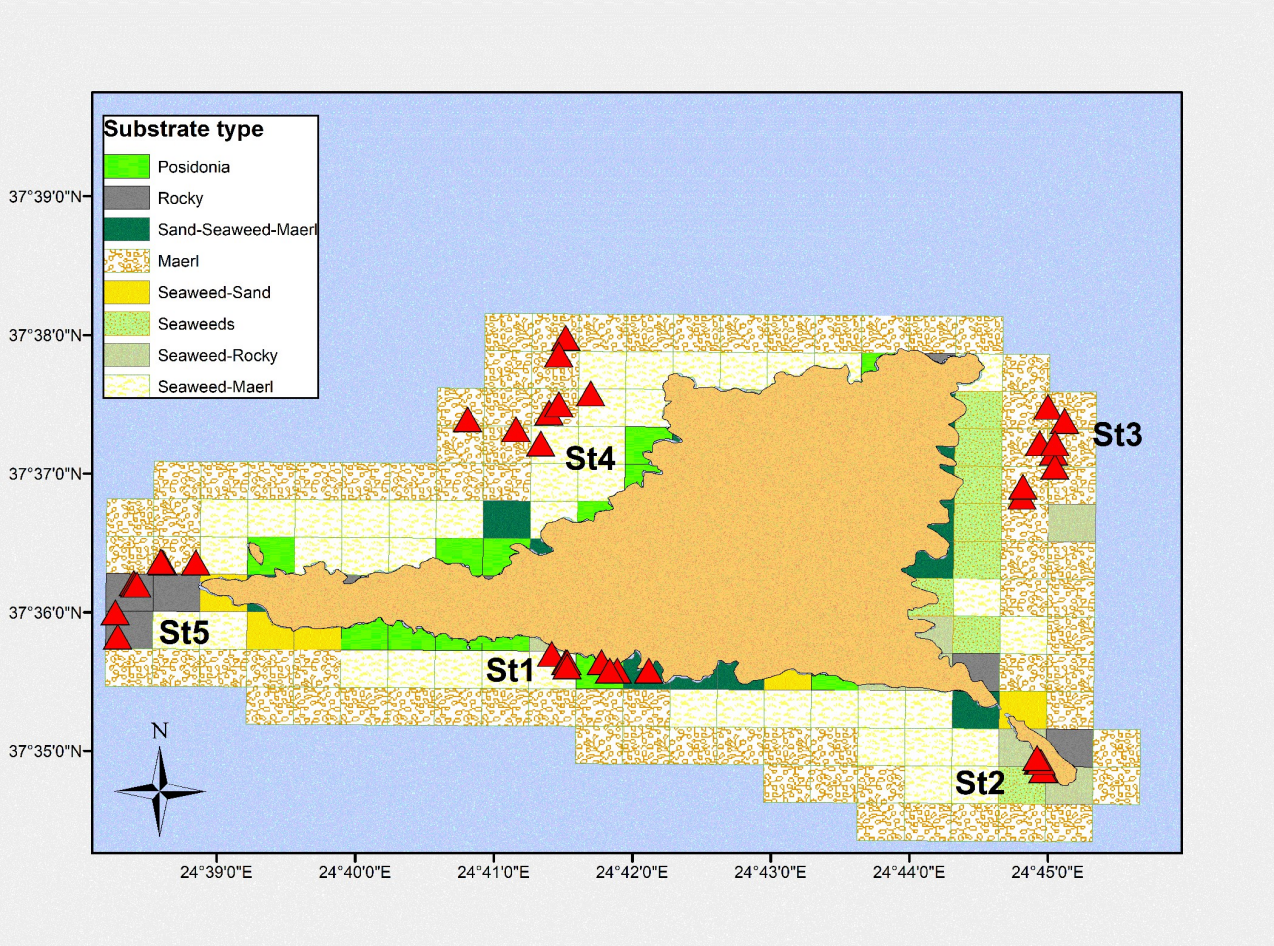
Substrate types around Gyaros island (sampling locations are depicted in red triangles). (Greece basemap reprinted from Kavadas et al., 2012 - https://doi.org/10.12681/mms.324 under a CC BY license, with permission from Stefanos Kavadas-HCMR, original copyright 2004).

Experimental fishing surveys have been carried out in five fixed locations around the island (Fig 2 and Table 1). The locations were selected applying NOAAs Sampling Design Tool (https://coastalscience.noaa.gov/project/sampling-design-tool-arcgis/), taking into account the depth strata and the types of bottom substrate. Samplings were carried out seasonally (four times per year: winter, spring, summer, autumn) during the period June 2018 –September 2020.

**Table 1:** Geolocations of sampling stations in Gyaros MPA during 2018–2020.

Station ID	Location name	Latitude (North)	Longitude (East)	Depth (m)	Substrate
St1	Fyllada	37 35 421 N	24 42 129 E	18	*Posidonia*/Rocky
St2	Glaronissi	37 34 860 N	24 45 015 E	17	*Posidonia*/Rocky
St3	Fournaki	37 37 486 N	24 45 117 E	98	Maerl/Sandy
St4	Colata	37 37 323 N	24 40 998 E	88	Maerl
St5	Fouis	37 36 367 N	24 38 612 E	47	Rocky

**Fig 3:**
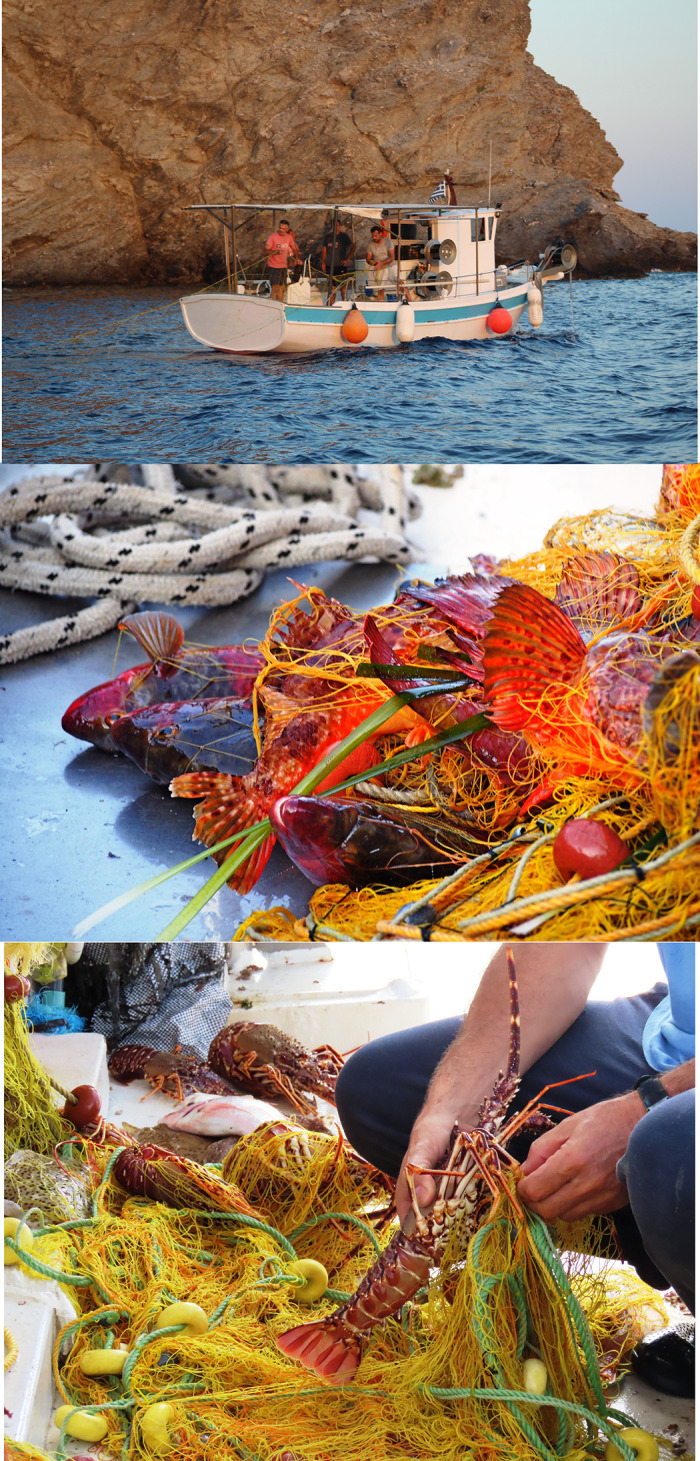
Sampling on board the F/V ‘Agia Trias’ in Gyaros island during September 2018.

Acquiring a permit for experimental fishing from the competent authority (Decentralized Administration of the Aegean) was a quite lengthy procedure, since numerous other entities had to be consulted prior to granting our request (e.g.: Coast guard, Directorate of Fisheries). Fishing trials were executed on board two chartered commercial fishing vessels from the nearby island of Syros: ‘Agia Trias’ and ‘Chryssoula’ of a local fishers’ family (Fig 3). Two full annual sampling cycles were completed, with a total of 40 fishing sets in 8 fishing excursions (2 years x 4 seasons x 5 stations = 40).

Prior to embarking into sampling trials, we conducted a round of questionnaire surveys with local fishers to identify which type of fishing would be of interest to them in the waters of Gyaros island. Trammel nets was the main outcome and as a result the fishing gears used were static trammel nets with a mesh size greater than 32 mm, lengths from 500 to 1000 meters and a height of around 2 meters. Nets were cast at late afternoon and retrieved early next morning at sunrise. Depths and substrate types over which fishing took place varied among the sampling stations.

### Sampling outside the MPA

#### Commercial fisheries data

The most detailed source of information comes from the EU Fisheries Data Collection Framework realized since 2003 in implementation of the EU Common Fisheries Policy (National Fisheries Data Collection Project (EPSAD) https://imbriw.hcmr.gr/national-fisheries-data-collection-project-epsad/ and https://datacollection.jrc.ec.europa.eu/documents/10213/1341570/Greece_Annual_Report_2019_Text.pdf/7654779a-9eb9-4d9b-ba55-c33343e05eef). Based on the aforementioned dataset, hosted in the HCMR database [8], we have analysed the data of the small-scale fishery (SSF) catches in the vicinity of Gyaros island. These data are based on monitoring the fishing activities by observers stationed on board fishing vessels. For our study we selected SSF vessels with similar characteristics as the ones of our sampling surveys:

- operating with trammel nets
- of mesh size > 30 mm
- net length of > 400m
- fishing depth between 5 and 105 meters
- during the period 2018–2020
- in the areas surrounding Gyaros island

#### Logbook of vessel used in the experimental trials

Another source of information came from the small-scale fishing vessel we have chartered to realize our experimental surveys. The logbooks of fishing sets conducted by this same vessel during 2017–2018 in the islands surrounding Gyaros MPA were analysed with the intention to compare against the fishing trials inside the Gyaros MPA. As a word of notion, official logbooks suffer from under-reporting of catches and especially non-commercial or low value species are usually absent. To this end, we focused only on commercial species, which were observed in our experimental fishing surveys as well. Nevertheless, the analysis was conducted in close collaboration with the fisher and can be considered a reliable source of information.

### Measurements and data collection

Operational characteristics of each fishing set included: date, time (casting and retrieving), exact geolocation, bottom depth, substrate type, length and height of fishing net, mesh size, wind direction/intensity and weather conditions.

All specimens were separated after their collection from the net, identified at species level and stored in the vessels’ freezer. Individual measurements and weights were taken at the HCMR’s laboratories. The measurements consisted of total length-TL (in mm) for fish (plus disc width-DW for skates/rays), mantle length-ML for cephalopods, and carapace length-CL for crustaceans, as well as a series of biological data such as: total and eviscerated weight, sex, maturity stage, gonad and liver weight. Life history data for each species were extracted after an exhaustive bibliographic review (see S1 File for a full list).

### Statistical analyses

Catch was measured both in numbers and weight of individuals. Biomass abundance was expressed in Catch weight Per Unit of effort-CPUEW (kg/1000 m of net) while nominal abundance was expressed as CPUEN (Catch number of individuals/1000m of net).

Multi-factor analysis of variance (ANOVA) was performed to compare abundance, size of specimens caught and diversity indices among sampling locations inside the MPA, as well as with catches from the commercial SSF fleet outside the MPA. Log-transformations were applied when necessary to transform skewed data so that they conform to normality [9].

Spatial density of key species inside the MPA was estimated by applying Generalized Additive Models (GAMs) [10]. The main assumption made is that the functional relationships between population density of marine species and explanatory variables are non-linear, and GAMs is a tool for addressing such issues. Implementation was realized through the mgcv package [11] in R v.4.0.3 [12]. In a GAM, the expected values of the response variable (biomass abundance CPUEW) were related to the predictor variables Z_m_ (substrate, depth, longitude, latitude) according to the following general formulation:

$$f(CPUEW)=c+\sum_{m}S_{m}(Z_{m_{i}}),$$

where f is the link function, c is the intercept, S_m_() is the one-dimensional smooth function of covariate Z_m_, and Z_mi_ is the value of covariate m for the i-th observation.

Spatial estimations of catch rates for the most common species, as well as the total catch, were derived in the form of gridded matrices for the area around Gyaros island using *predict*.*gam*() function of the mgcv package. The method is, in brief, an extrapolation of biomass abundance (CPUEW) from areas of known (surveyed) abundance to areas where only some environmental characteristics are known (substrate, depth, longitude, latitude), assuming that these are good predictors of abundance. The marine region (37^o^34’20”– 37^o^38’10” North, 24^o^38’12”–24^o^45’40” East) was gridded in a spatial resolution of 0.01 x 0.01 of a degree, concluding to a total of 180 grid cells (land excluded). Each one of these cells was assigned the corresponding values for each of the model parameters (e.g.: depth, substrate type, latitude, longitude). With the intention to visualize the results, these matrices of gridded spatial predictions, were stored as Geographical Information System (GIS) raster datasets and mapped using ESRI’s ArcMap desktop GIS software.

Faunistic similarities among the different sampling sites in the protected area were estimated using the Bray-Curtis similarity index [13]. Similarity matrices were constructed from the abundance matrices and multivariate analyses i.e.: hierarchical clustering analysis (CLUSTER) and Non- metric Multi-Dimensional Scaling (MDS) were performed [14]. To assess which species were responsible for the dissimilarities between the sampling sites, the two-way similarity percentages (SIMPER) non- parametric routine was conducted [15]. Species richness, Shannon Wiener Ή [16] was calculated for each sampling site based on abundance data for all sampling seasons. Finally, Abundance Biomass Comparison (ABC) curves were constructed for each sampling site at all seasons. All diversity analyses were conducted using the PRIMER-6 software package [17].

## Results

### Species composition and abundance

A total of 75 species/taxa have been identified in the surveys, the most abundant in terms of biomass being: parrotfish-*Sparisoma cretense*, red scorpionfish-*Scorpaena scrofa*, common spiny lobster-*Palinurus elephas*, red porgy-*Pagrus pagrus*, little tunny–*Euthynnus alltetteratus*, Mediterranean moray-*Muraena helena*, lesser spotted dogfish-*Scyliorhinus canicula*, forkbeard-*Phycis phycis*, Surmullet-*Mullus surmuletus*, common cuttlefish-*Sepia officinalis* and common pandora-*Pagellus erythrinus*. The full list of species/taxa is provided in Table 2.

**Table 2:** List of species/taxa identified in the experimental fishing trials with nets in Gyaros island (sorted by contribution to total biomass—catch in grams (g) and in number of individuals (Nb)).

Species/taxon	English name	Catch (in g)	Catch (in Nb)	Species/taxon	English name	Catch (in g)	Catch (in Nb)
*Sparisoma cretense*	Parrotfish	106737	254	*Dasyatis pastinaca*	Common stingray	885	1
*Scorpaena scrofa*	Red scorpionfish	35810	96	*Stylocidaris affinis*	Red sea urchin	860	85
*Palinurus elephas (Fabricius*, *1787)*	Common spiny lobster	24336	32	*Dactylopterus volitans*	Flying gurnard	819	2
*Pagrus pagrus*	Red porgy	16230	33	*Sphyraena viridensis*	Yellowmouth barracuda	730	1
*Euthynnus alletteratus*	Little tunny	11900	12	*Chelon labrosus*	Thicklip grey mullet	700	2
*Muraena helena*	Mediterranean moray	11066	7	*Squalus blainville*	Longnose spurdog	700	1
*Scyliorhinus canicula*	Lesser spotted dogfish	10233	44	*Torpedo marmorata*	Marbled electric ray	691	2
*Phycis phycis*	Forkbeard	9632	17	*Dentex dentex*	Common dentex	650	1
*Mullus surmuletus*	Surmullet	9442	38	*Diplodus annularis*	Annular seabream	572	10
*Sepia officinalis*	Common cuttlefish	8633	26	*Labrus merula*	Brown wrasse	552	1
*Pagellus erythrinus*	Common pandora	7719	28	*Squalus acanthias*	Picked dogfish	543	2
*Scorpaena porcus*	Black scorpionfish	6884	36	*Trachurus trachurus*	Atlantic horse mackerel	490	2
*Spondyliosoma cantharus*	Black seabream	6747	31	*Symphodus tinca*	East Atlantic peacock wrasse	479	4
*Epinephelus costae*	Goldblotch grouper	5333	8	*Spicara maena*	Blotched picarel	452	6
*Diplodus vulgaris*	Common two-banded seabream	5292	45	*Hexaplex trunculus*	Banded dye-murex	401	29
*Raja clavata*	Thornback ray	5057	4	*Calliactis parasitica*	‘Parasitic’ anemone	369	38
*Siganus luridus*	Dusky spinefoot	4604	34	*Raja polystigma*	Speckled ray	364	1
*Dardanus calidus*	Red hermit crab	4588	64	*Codium bursa*	Green sponge ball	300	11
*Pseudocaranx dentex*	White trevally	3351	5	*Stephanolepis diaspros*	Reticulated leatherjacket	275	4
*Raja radula*	Rough ray	2848	6	*Scorpaena notata*	Small red scorpionfish	243	1
*Scyllarides latus*	Mediterranean slipper lobster	2518	4	*Murex brandaris*	Purple dye murex	220	4
*Trachinus radiatus*	Starry weever	2293	4	*Serranus cabrilla*	Comber	163	5
*Diplodus puntazzo*	Sharpsnout seabream	2018	3	*Aulopus filamentosus*	Royal flagfin	163	1
*Sarpa salpa*	Salema	1942	4	*Chelidonichthys lastoviza*	Streaked gurnard	144	1
*Sciaena umbra*	Brown meagre	1864	3	*Zeus faber*	John dory	123	1
*Epinephelus marginatus*	Dusky grouper	1687	3	*Dardanus arrosor*	Striated hermit crab	100	3
*Uranoscopus scaber*	Stargazer	1617	6	*Spicara flexuosa*	Blotched picarel	87	1
*Labrus mixtus*	Cuckoo wrasse	1576	2	*Boops boops*	Bogue	50	1
*Calappa granulata*	Shamefaced crab	1500	25	*Phallusia mammillata*	White warty seasquirt	50	1
*Oblada melanura*	Saddled bream	1337	4	*Apogonichthyoides nigripinnis*	Bullseye	35	2
*Sarcotragus spinosulus*	Black leather sponge	1300	1	*Bolma rugosa*	Rough turbo	20	1
*Serranus scriba*	Painted comber	1121	8	*Ascidia spp*.	Ascidians	20	4
*Raja miraletus*	Brown ray	1081	3	*Echinoidea*	Sea urchins	20	1
*Phycis blennoides*	Greater fork-beard	1008	4	*Cnidaria*	Jellyfish	20	1
*Cidaris cidaris*	Pencil urchin	955	88	*Maerl*	calcareous red algae	20	1
*Sphyraena sphyraena*	European barracuda	953	2	*Anthias anthias*	Swallowtail seaperch	18	1
*Scomber colias*	Atlantic chub mackerel	898	2	*Maja goltziana*	Spiny spider crab	15	1
				**Grand Total**		**334452**	**1220**

Sorted by Phylum, Chordata prevailed (86%), followed by Arthropoda and Mollusca, with ray-finned fish—Actinopterygii (79.8%), Malacostraca (9.5% e.g.: lobsters, crabs) and elasmobranchs -Elasmobranchii (6.7%—sharks and rays) comprising the vast majority of the catch. Most species/taxa were observed during summer and autumn and during 2019 (Table 3).

**Table 3:** Number of species/taxa observed by season and year in the experimental fishing trials with nets in Gyaros island.

Year	2018		2019	2020	Total
**Nb of species/taxa**	41		53	39	75
**Season**	winter	spring	summer	autumn	Total
**Nb of species/taxa**	37	29	48	40	75

Significant differences were identified among locations both in the composition of catch, as well as the biomass and abundance (see S1 File for detailed results). This was an effect of the diverse depths and substrates characterizing each sampling station. The shallow southern stations (St1, St2), over *Posidonia* meadows, were dominated by the parrotfish-*S*. *cretense*, while the deeper locations, over maerl beds, (St3, St4) hosted more common spiny lobsters-*P*. *elephas*. Finally, St5, over a rocky substrate, showed a quite diverse species synthesis (S1 and S2 Tables in S1 File). Another interesting finding was the lower number of species over maerl beds (St3, St4), compared to the locations over *Posidonia* meadows (St1, St2) (S1 Table in S1 File).

**Fig 4:**
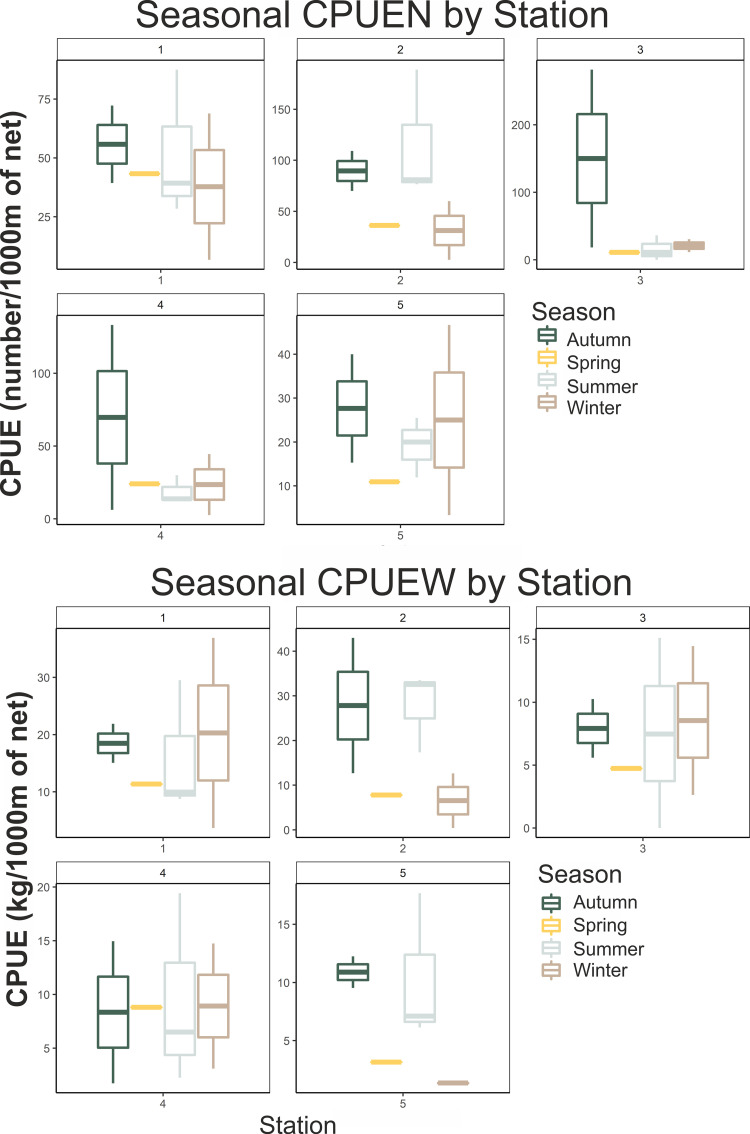
Box-plots of seasonal total species abundance in the five sampling locations around Gyaros island (top: CPUEW in kg/1000m of trammel net; bottom: CPUEN in number of individuals/1000m of trammel net).

Analysing abundance (in numbers) and biomass (in weight) data by season, such differences became evident (Fig 4). Results of an ANOVA test, with Season and Station as driving factors of biomass, revealed that both factors had an effect on total species biomass (Table 4). In contrast, there was no significant annual effect for the whole area catches, although such differences were apparent for some of the sampling locations (S3 Table in S1 File).

**Table 4:** Results of an ANOVA test on the effects of Season and Station on biomass (*: significant, at the 0.05 level).

Factors on CPUEW	Df	Sum Sq	Mean Sq	F value	Pr(>F)
**SEASON**	3	344.4	114.82	1.299	0.0291*
**STATION**	4	1131.2	282.8	3.201	0.0256*
**Residuals**	32	2827.5	88.36		

**Fig 5:**
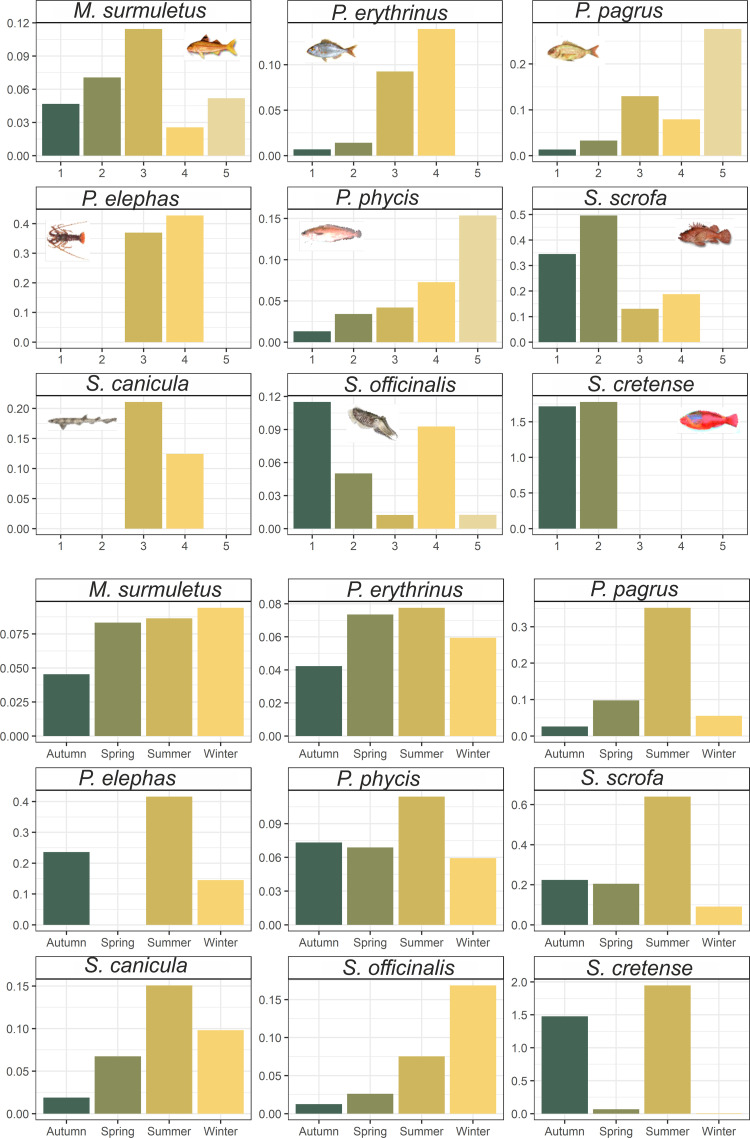
Biomass estimate for nine common species in the five sampling locations around Gyaros island (top) and among seasons (bottom) (CPUEW in kg/1000m of trammel net).

A more detailed analysis was carried out for nine abundant species of commercial interest that had a continuous presence throughout seasons/years: *M*. *surmuletus*, *S*. *scrofa*, *S*. *canicula*, *S*. *officinalis*, *S*. *cretense*, *P*. *erythrinus*, *P*. *pagrus*, *P*. *elephas and P*. *phycis* (Fig 5). Results of an ANOVA test, with substrate, year, season and depth as driving factors of biomass, revealed that substrate was the main driver for *S*. *scrofa*, *S*. *canicula* and *S*. *cretense*, while *M*. *surmuletus* varied among years (S4 Table in S1 File).

### Spatial density–suitable habitat

**Fig 6:**
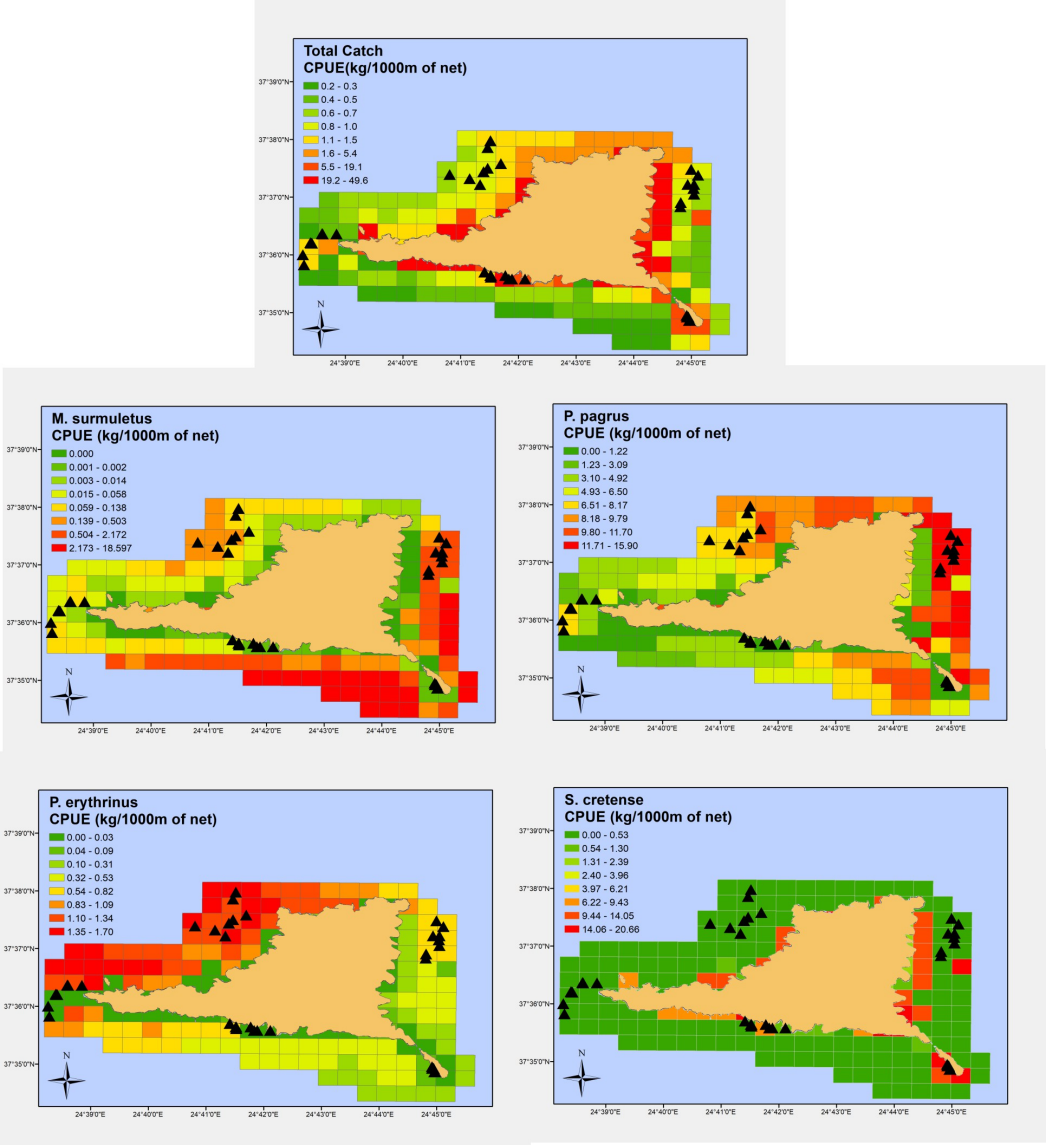
Spatially predicted relative abundance (kg of fish/1000 m of trammel net) for Total catch (top), surmullet (mid-left), red porgy (mid-right), common Pandora (bottom-left) and parrotfish (bottom-right) around Gyaros MPA (sampling locations are depicted in black triangles). (Greece basemap reprinted from Kavadas et al., 2012 - https://doi.org/10.12681/mms.324 under a CC BY license, with permission from Stefanos Kavadas-HCMR, original copyright 2004).

**Fig 7:**
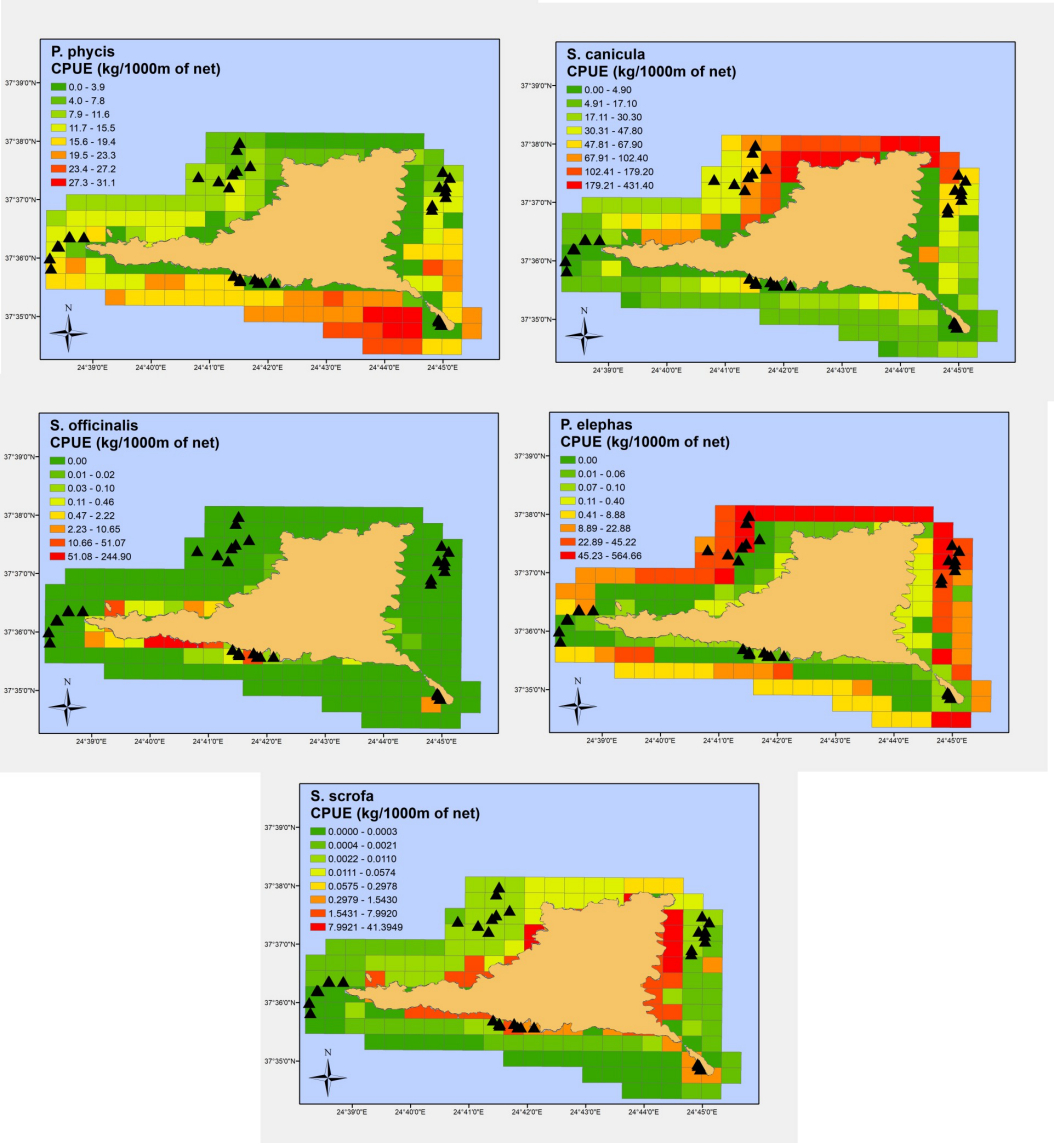
Spatially predicted relative abundance (kg of fish/1000 m of trammel net) for forkbeard (top-left), lesser spotted dogfish (top-right), common cuttlefish (mid-left), common spiny lobster (mid-right) and red scorpionfish (bottom) around Gyaros MPA (sampling locations are depicted in black triangles). (Greece basemap reprinted from Kavadas et al., 2012 - https://doi.org/10.12681/mms.324 under a CC BY license, with permission from Stefanos Kavadas-HCMR, original copyright 2004).

Results of the GAM analyses on the nine most common commercial species indicated the most influential predictors of biomass (Table 5). Figs 6 and 7 show prediction maps representing a potential or suitable habitat, as indicated by the linkage between predictor variables (substrate, depth, longitude, latitude) and fishing success at specific locations (CPUEW).

**Table 5:** Results of GAM analyses on the effects of substrate, depth, longitude and latitude on the biomass of the most common commercial species observed in Gyaros MPA (**: highly significant; *: significant,—: not significant).

Species	substrate	depth	longitude	latitude
***Mullus surmuletus***	**	**	**	**
***Pagellus erythrinus***	**	-	-	-
***Pagrus pagrus***	**	**	**	-
***Palinurus elephas***	**	-	-	-
***Phycis phycis***	**	**	**	**
***Scorpaena scrofa***	**	-	-	*
***Scyliorhinus canicula***	**	-	-	*
***Sepia officinalis***	**	**	**	**
***Sparisoma cretense***	**	-	-	-
***Total catch***	**	-	-	-

In terms of total catch, substrate type was the main driver; areas adjacent to the coast covered by *Posidonia* meadows or seaweeds were the more prolific (Fig 6 –top). At species level, surmullet and the common pandora were more abundant over maerl beds; the former in the south-eastern side of the island (Fig 6 –mid left) and the latter in the northwest side (Fig 6 –bottom left). The red porgies were also associated with maerl beds, however they resided in deeper waters (Fig 6 –mid right). The parrotfish, being the most abundant species, was exclusively found over *Posidonia* meadows and seaweeds and at very shallow depths by the coast (Fig 6 –bottom right). The forkbeard showed a clear preference for deeper waters in the southeast region (Fig 7 –top left) while the lesser spotted dogfish was restricted to the northern part of the island over maerl beds (Fig 7 –top right). The common cuttlefish had a very limited spatial distribution, over shallow coastal waters covered by vegetation in the southern part of the island (Fig 7 –mid left). The common spiny lobster’s abundance was clearly related to the presence of coralligenous habitats, with its spatial distribution being almost identical with the distribution of maerl beds (Fig 7 –mid right). Finally, the red scorpionfish was found in great abundance throughout the shallow coastal zone around Gyaros (Fig 7 –bottom). Almost all estimates, were accompanied by a high uncertainty for the grid cells at deeper waters and the northern part; this should be attributed to the limited samples over waters with depths > 100 m and the northern marine region (see S1 and S2 Figs in S1 File for maps depicting standard errors around the estimates).

### Species diversity

**Fig 8:**
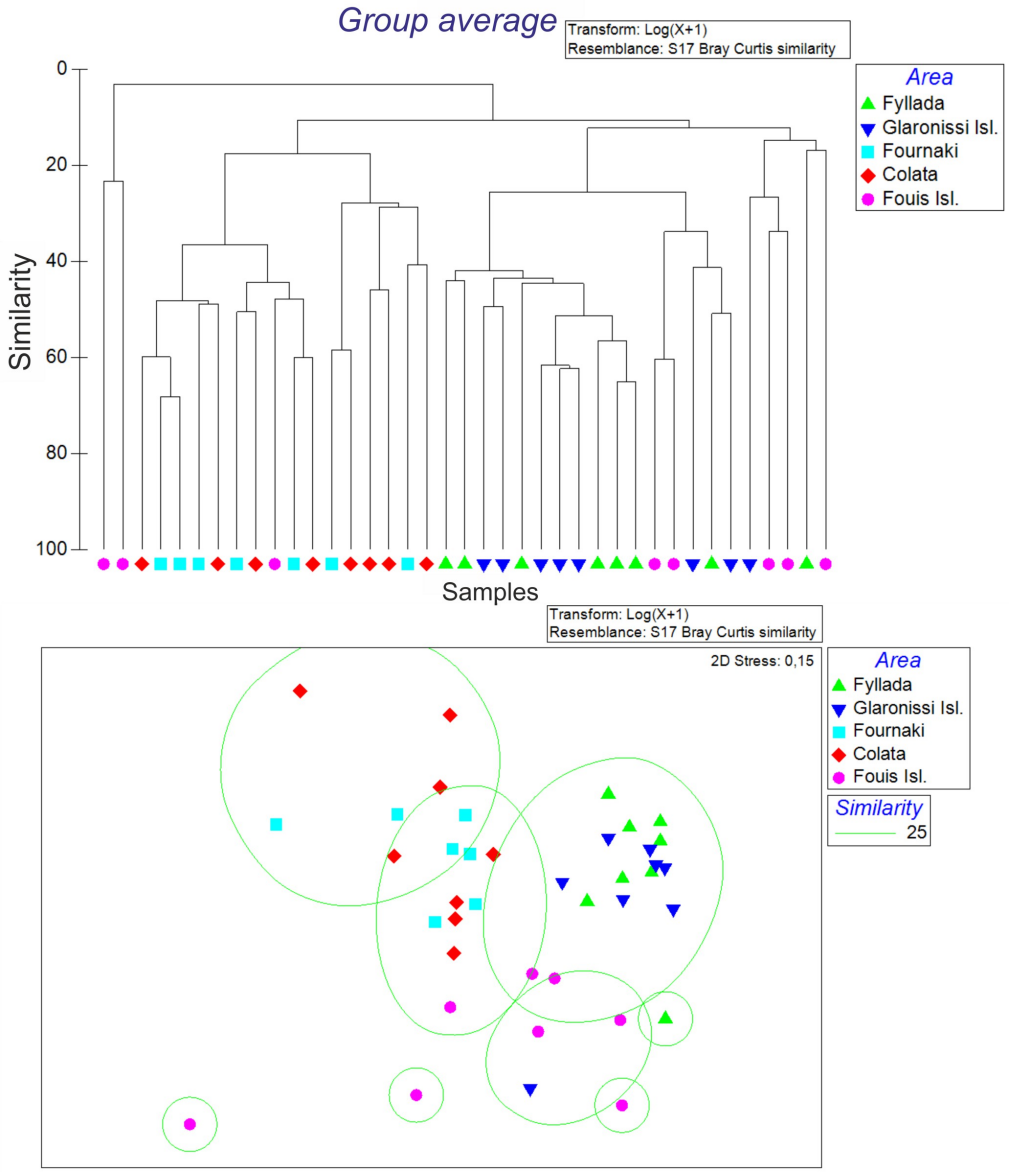
Similarity of the studied locations in Gyaros MPA, based on catch abundance, demonstrated in a dendrogram derived from hierarchical clustering analysis (top) and in a Non-metric Multi-Dimensional Scaling plot (bottom).

Similarity analysis showed three distinct groups between the sampling areas (Fig 8). Station 1- Fyllada and Station 2-Glaronissi were grouped together, at a similarity level of 34.09%, while Station 3-Fournaki and Station 4-Colata were grouped at a similarity level of 31.78%. Station 5-Fouis stood by itself having a dissimilarity level of over 87% with all the other areas. St 5-Fouis was also the sampling location with the most inconsistent level of similarity among the sampling seasons for both abundance (16.28% similarity) and biomass (18.40% similarity).

SIMPER analysis revealed that the dominant species responsible for the dissimilarities between the different sampling locations in terms of abundance (S5 Table in S1 File) were *S*. *cretense*, *S*. *canicula*, *S*. *scrofa* and *Dardanus callidus* (red hermit crab) in the case of St 1-Fyllada and St 2-Glaronissi and *S*. *canicula*, *Cidaris cidaris* (pencil urchins), *P*. *erythrinus* and *P*. *elephas* in the case of St 3-Fournaki and St 4-Colata (S6 Table in S1 File).

**Fig 9:**
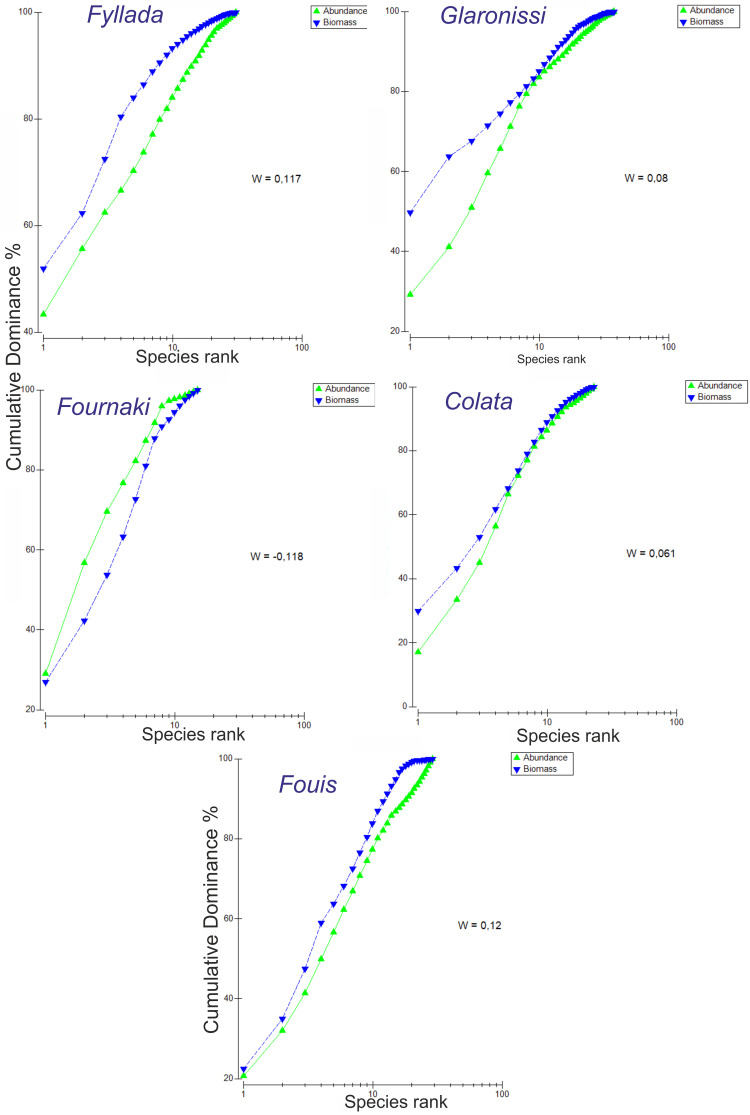
ABC plots calculated for each sampling site in the Gyaros MPA.

The Abundance Biomass Comparison-ABC dominance plots followed the pattern of initial stability (biomass over abundance curve–[18]), in all sampling sites except from the case of St 3-Fournaki sampling site where the abundance curve is over the biomass curve (Fig 9).

### Demography of catches

Analysis of biological data revealed that most species observed in Gyaros MPA were represented by quite large and mature individuals. Table 6 displays length statistics for the measured species, as well as the proportion below MLS-Minimum Landing Size (for those regulated by an MLS—currently MCRS (Minimum Conservation Reference Size), the length at which at least 50% of the population is considered to be mature (L_50_ mat) and the proportion below this threshold. All life history values in Table 6 were extracted from relevant studies after an exhaustive bibliographic review (see S1 File).

**Table 6:** Length statistics for the species observed in Gyaros MPA (in mm). Species ordered by sample size. Results can be considered meaningful only for the 15 species having a sample size > = 10. (green: no specimens below MLS or L_50_mat; yellow: less than 50% of specimens below MLS or L_50_mat; red: more than 50% of specimens below MLS or L_50_mat).

Species	Mean	Median	Min	Max	Sample size	MLS	% below MLS	L_50_ mat	% below L_50_ mat
*Sparisoma cretense*	291.7	293.5	213	395	254			155	0
*Scorpaena scrofa*	265.4	263	135	444	93			249	37,9
*Diplodus vulgaris*	195	189.5	152	255	45	180	24.4	175	17.8
*Scyliorhinus canicula*	414.2	420.5	346	474	44			399	18.2
*Mullus surmuletus*	269	273	155	335	38	110	0	153	0
*Scorpaena porcus*	207.5	205	144	264	36			175	13.9
*Siganus luridus*	198.4	201	159	232	34			142	0
*Pagrus pagrus*	312.2	301	187	518	33	180	0		
*Spondyliosoma cantharus*	234.9	228.5	175	315	32			178	0.3
*Pagellus erythrinus*	281.2	281	206	360	28	150	0	134	0
*Sepia officinalis*	144.8	142	119	178	26			90	0
*Palinurus elephas*	127.8	100	49	406	25	90	36	100	**48**
*Phycis phycis*	348.1	345	236	490	16			310	37.5
*Euthynnus alletteratus*	428.9	431	390	469	12			420	33.3
*Diplodus annularis*	152.3	154	134	171	10	120	0	106	0
*Epinephelus costae*	374.8	375.5	266	490	8	450	**87.5**	350	37.5
*Serranus scriba*	210.9	213	198	224	8			93	0
*Muraena helena*	874	898	646	1050	7			760	28.6
*Raja radula*	415.7	433.5	329	462	6			565	0
*Uranoscopus scaber*	244.5	244.5	213	275	6			118	0
*Pseudocaranx dentex*	382.4	375	348	415	5				
*Spicara maena*	172	180	132	187	5			131	0
*Oblada melanura*	278.3	288	231	306	4				
*Phycis blennoides*	278	245	235	387	4				
*Raja clavata*	607	607	373	841	4				
*Sarpa salpa*	306.5	313.5	240	359	4				
*Scyllarides latus*	95.3	89	77	126	4				
*Serranus cabrilla*	142.7	142	125	161	4			132	**50**
*Stephanolepis diaspros*	159.3	171	116	179	4			80	0
*Symphodus tinca*	219	220	198	238	4			100	0
*Trachinus radiatus*	370.5	393	281	415	4			243	0
*Diplodus puntazzo*	337.3	350	307	355	3	180	0		
*Epinephelus marginatus*	324.7	305	279	390	3	450	**100**	367	**66.7**
*Raja miraletus*	410.7	427	375	430	3			418	33.3
*Sciaena umbra*	352.7	340	240	478	3				
*Scomber colias*	317.3	309	301	342	3	180	0	346	**100**
*Apogonichthyoides nigripinnis*	99	99	96	102	2				
*Chelon labrosus*	416	416	370	462	2				
*Dactylopterus volitans*	338	338	316	360	2				
*Labrus mixtus*	376.5	376.5	372	381	2			152	0
*Sphyraena sphyraena*	523.5	523.5	501	546	2			276	0
*Squalus acanthias*	363.5	363.5	243	484	2				
*Torpedo marmorata*	256.5	256.5	235	278	2				
*Trachurus trachurus*	298	298	296	300	2	150	0	220	0
*Anthias anthias*	137	137	137	137	1				
*Aulopus filamentosus*	263	263	263	263	1				
*Dasyatis pastinaca*	490	490	490	490	1				
*Dentex dentex*	372	372	372	372	1			230	0
*Labrus merula*	340	340	340	340	1				
*Maja goltziana*	39	39	39	39	1				
*Raja polystigma*	390	390	390	390	1				
*Scorpaena notata*	245	245	245	245	1			92	0
*Sphyraena viridensis*	615	615	615	615	1			625	0
*Spicara flexuosa*	185	185	185	185	1			103	0
*Squalus blainville*	560	560	560	560	1				
*Trigloporus lastoviza*	247	247	247	247	1			139	0
*Zeus faber*	200	200	200	200	1			254	**100**

Only 15 species had an adequate sample size (>10) allowing to infer useful outcomes. The majority of these individuals were above both regulatory (MLS) and biological thresholds (L_50_ mat). The zero proportion of undersized fish for the Mediterranean parrotfish, the striped red mullet, the black seabream and the common pandora are some quite impressive results. For the remaining species, a ‘flag’ of concern can be raised for the two groupers (*Epinephelidae*), since 90% of specimens were below the set MLS.

For the nine abundant species of commercial interest that had a continuous presence throughout seasons/years: *M*. *surmuletus*, *S*. *scrofa*, *S*. *canicula*, *S*. *officinalis*, *S*. *cretense*, *P*. *erythrinus*, *P*. *pagrus*, *P*. *elephas and P*. *phycis*, we employed more detailed analysis to detect plausible variations in size by sampling location, season and year (Table 7).

**Table 7:** Results of an ANOVA test on the effects of Season, Year and Station on size of the most common commercial species observed in Gyaros MPA (**: highly significant; *: significant, -: not significant).

Species	Season	Year	Station
***Mullus surmuletus***	-	-	**
***Pagellus erythrinus***	-	-	-
***Pagrus pagrus***	-	-	-
***Palinurus elephas***	**	**	-
***Phycis phycis***	-	-	-
***Scorpaena scrofa***	-	-	**
***Scyliorhinus canicula***	**	-	-
***Sepia officinalis***	-	-	-
***Sparisoma cretense***	**	**	-

Sampling location affected the size of the captured surmullets (*M*. *surmuletus*) and red scorpionfish (*S*. *scrofa*); larger specimens were observed in the deeper stations, in contrast to the shallow ones. The size of the common spiny lobster (*P*. *elephas*) and the parrotfish (*S*. *cretense*) was dependent on season and year; common spiny lobsters being larger during winter and parrotfish smaller. Lesser spotted dogfish (*S*. *canicula*) size was highly seasonal, with larger specimens being captured during summer and smaller during winter.

Sex ratio was in favor of females for the red scorpion fish, the surmullet, the red porgy, the common pandora and the lesser spotted dogfish, while the opposite was apparent for the common spiny lobsters and the common cuttlefish. Sex ratio did not statistically deviate from 1:1 for greater forkbeard and parrotfish. Significant seasonal fluctuations in sex ratios were observed for common spiny lobsters, forkbeard, lesser spotted dogfish and common cuttlefish, indicating spawning aggregations by sex (S7 Table in S1 File).

### In and out the MPA–a comparison

#### Abundance and richness

During the period 2018–2020, a set of six small-scale fishing vessels from the neighboring islands, with a total of 13 trips, fulfilled the criteria set (operational characteristics similar to the ones employed inside the MPA—see Materials & Methods and S0 Table in S1 File). The average standardized catch rate (expressed as CPUEW) of the aforementioned SSF vessels was 7.9 kg per 1000 meters of net deployed (min 2.7, max 22.7)–compared to the 10.95 kg/1000m of net for the experimental fishing surveys inside the MPA. A total of 58 species/taxa were recorded (S8 Table in S1 File). More than half of the catches comprised of six species: common cuttlefish-*S*. *officinalis*, thornback ray-*R*. *clavata*, red scorpionfish-*S*. *scrofa*, common spiny lobster-*P*. *elephas*, smoothhound-*Mustelus mustelus*, and parrotfish-*S*. *cretense*. It must be noted here that the on-board observers implementing the EU Fisheries Data Collection Framework (DCF) in Greece record all captured species and these results do not lack non-commercial or discarded species. Commercial or non-commercial categorization for each species was defined based on the observations onboard fishing vessels during the implementation of DCF throughout the years [19]. In the experimental surveys, a total of 75 species were recorded; 3 of them making up more than 50% of the catch in weight: *S*. *cretense*, *S*. *scrofa* and *P*. *elephas*. Fish (bony fish and chondrichthyans) dominated the catches (Table 8); non-commercial taxa comprised 10% of the catch inside the MPA.

**Table 8:** Catch composition by major faunistic category in and out the MPA when comparing experimental surveys with commercial fishing catches.

Faunistic category	Outside the MPA	Inside the MPA
**Bony fish**	75.86%	62.67%
**Chondrichthyans**	10.34%	10.67%
**Crustaceans**	8.62%	8.00%
**Molluscs**	0.00%	5.33%
**Echinodermata**	0.00%	4.00%
**Tunicates**	0.00%	2.67%
**Cnidarian**	0.00%	2.67%
**Porifera**	0.00%	1.33%
**Cephalopods**	5.17%	1.33%
**Chlorophyta**	0.00%	1.33%

Analysis of size data indicated that most species captured by the commercial small-scale fishing fleet in the surrounding islands were mostly large mature individuals. Apparently, this is a result of the specific fishing gear characteristics we used in our analyses; this being trammel net with a quite large mesh size (>30 mm) allowing for undersized fish to escape capture. S9 Table in S1 File displays length statistics for the measured species, as well as the proportion below MLS-Minimum Landing Size (for those regulated by an MLS).

Furthermore, from the logbooks of fishing sets conducted by the same vessel used in the experimental surveys we managed to extract a total of 38 fishing sets with similar operational characteristics (trammel nets of mesh size >30mm, net length > 2000 m). Only species of commercial interest were included in these records. A total of 15 species were landed and sold at the local markets; red scorpionfish and parrotfish dominated, comprising almost 50% of total catch (S10 Table in S1 File).

### Diversity

**Fig 10:**
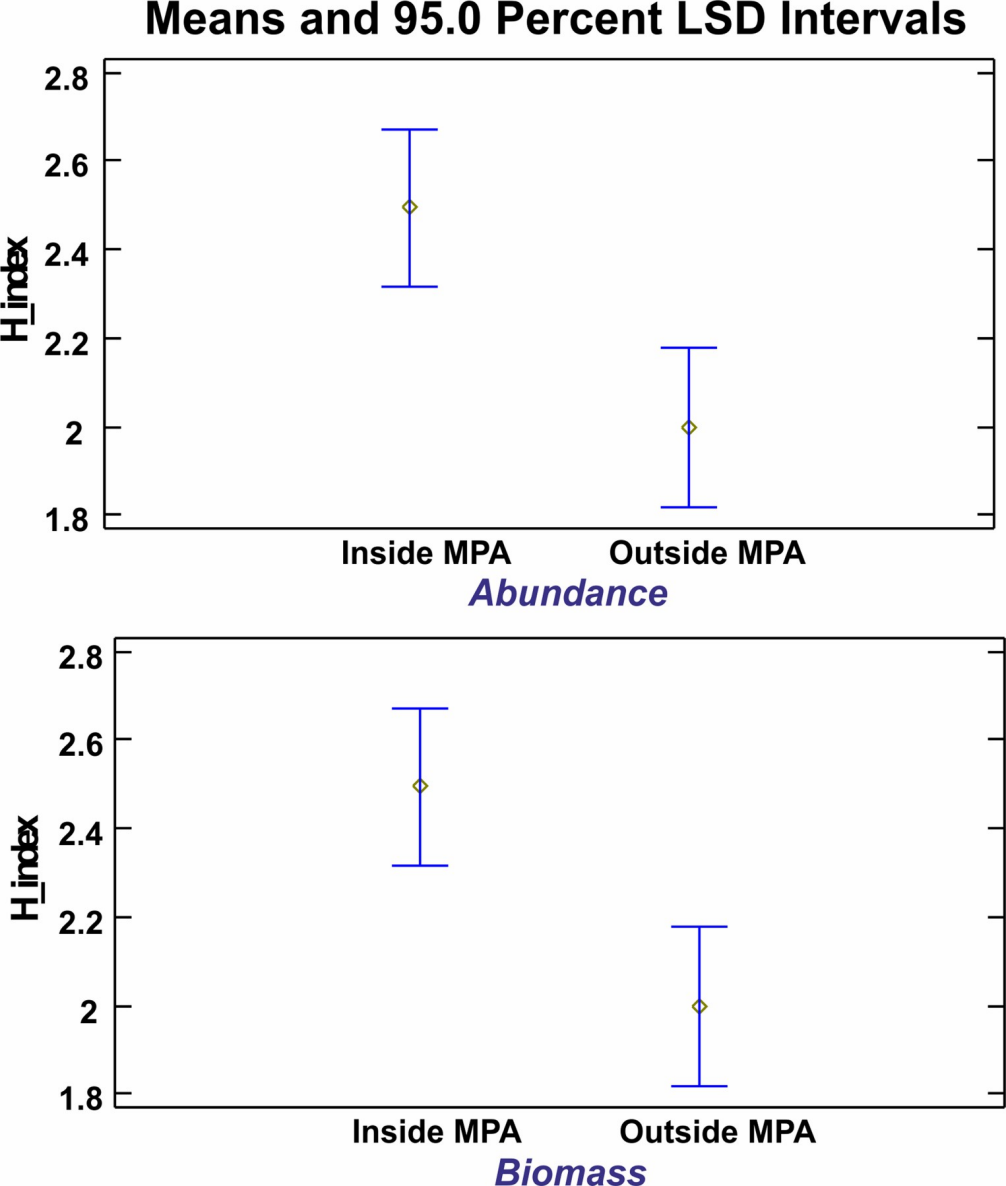
Means plot of H’ indices in and out the MPA.

Community functioning based on diversity indices in and out of the MPA was assessed based on the comparison of the Shannon–Wiener H’ indices, both for biomass (in weight) and abundance (in numbers). Results of a GLM ANOVA investigating the effect of area (in/out) on the H’ index revealed statistically significant differences (*p* <0.05) among areas with higher values of the index inside the MPA (Fig 10). It is crucial to highlight here that the Shannon -Wiener Index is used only as a comparison tool and not as an ecological status index, as the sampling methodology with static trammel nets is characterized by high selectivity; as a result, the number of species used for the analysis may not be reflecting the actual community structure of the area sampled.

### Demography

For the species measured for morphometrics and having an adequate sample size to allow comparisons, we calculated the average length in and out of the MPA, comparing data from the experimental surveys (in MPA) with the commercial fishing observations (out of MPA). 24 out of 36 species were larger inside the MPA, and 11 out of 36 were larger outside the MPA (S11 Table in S1 File).

For the nine most abundant species of commercial interest we have employed a series of ANOVA tests, to assess if these differences are actually statistically significant. Results suggested that 7 out of these 9 species were indeed larger inside the MPA (Table 9).

**Table 9:** Average length (in mm) of the nine most abundant commercial species captured in and out of Gyaros MPA and results of an ANOVA test on the effects of area (In–Out of MPA) on size (**: highly significant; *: significant, n.s.: not significant).

Species	Sample size	In	Out	% difference In-Out	ANOVA
***Pagrus pagrus***	40	312.2	230.1	36%	**
***Phycis phycis***	21	348.1	266.2	31%	*
***Mullus surmuletus***	77	269.0	215.3	25%	**
***Scorpaena scrofa***	150	265.4	219.1	21%	**
***Palinurus elephas***	40	127.8	107.3	19%	n.s.
***Sepia officinalis***	87	144.8	128.5	13%	**
***Sparisoma cretense***	278	291.7	261.6	11%	**
***Scyliorhinus canicula***	69	414.2	389.8	6%	**
***Pagellus erythrinus***	35	281.2	287.3	-2%	n.s.

### Alien species

During the program’s samplings, three (3) alien fish species were collected; bullseye-*Apogonichthyoides nigripinnis*, dusky spinefoot-*Siganus luridus* and reticulated leatherjacket-*Stephanolepis diaspros* accounting for 1.5% of the species caught in weight and 3% in numbers. No alien species were observed outside the MPA.

Two individuals of *A*. *nigripinnis* were caught during autumn 2018 in St 2—Glaronissi and four individuals of *S*. *diaspros* were caught in St 1—Fyllada in almost all sampling cruises except from the winter samplings. The most common representative among the three alien species was *S*. *luridus*, with 34 individuals caught in St1—Fyllada and St2—Glaronissi sampling sites in all sampling cruises except those taken place during winter.

Notable that all alien species specimens were caught in sampling sites with shallow waters; none was observed in depths > 20m.

### Assessment of MPA status

Based on the experimental fishing trials with static trammel nets inside the MPA and the comparison with similar activities outside the MPA, a series of four indicators were evaluated towards assessing the MPA functioning.

- Species diversity index
- Species relative abundance/biomass index
- Key predator species (i.e.: *Epinephelidae*) abundance
- Alien fish abundance

The results are summarized in Table 10.

**Table 10:** Indicators for assessing MPA status in Gyaros.

Indicators	Parameter	Result
***Species diversity index (in and out of the MPA)***	Shannon–Wiener H’ index	$\underline{\mathrm{Positive}}:$ Stat. significant higher values of the index inside the MPA
***Species relative biomass index (in and out of the MPA)***	CPUEW (kg/1000 m of trammel net)	$\underline{\mathrm{Positive}}:$ CPUEW inside the MPA was higher (38% on average—up to 9 times higher for certain species)
***Key predator species (i*.*e*. *Epinephelidae) abundance/size***	CPUEW (kg/1000 m of trammel net)	$\underline{\mathrm{Positive}}:$ CPUEW inside the MPA was higher (as much as 3 times higher)
	Total length	$\underline{\mathrm{Negative}}:$ Majority of specimens inside MPA smaller than length at maturity (immature)
		$\underline{\mathrm{Positive}}:$ Average size of groupers larger inside MPA (marginal difference 6%)
***Alien fish abundance***	CPUEW (kg/1000 m of trammel net)	3 species observed—1.5% in numbers and 3% in weight of catch.
		$\underline{\mathrm{Negative}}:$ No alien species observed outside the MPA

Overall, in view of the primary goal of the MPA to protect biodiversity, the MPA appears to be effective and functioning, since both species diversity and abundance is higher inside the protected area. However, its performance may still not be considered as optimal, as this is indicated by the large proportion of undersized key predators (e.g. groupers), although somewhat larger than the ones residing outside the MPA.

## Discussion

MPAs are means to safeguard marine biodiversity (species, habitats and processes), and are considered by some as the optimal tool for managing marine resources ([1] and references therein). Successful stories of MPA implementation in the Mediterranean include Taza National Park-Algeria [20], Côte Bleue Marine Park-France [21], Torre Guaceto-Italy [22] and Gokova bay-Turkey [23]. As a rule, site protection or low fishing pressure have been proven to be beneficial both for abundance and biomass as well as species richness [24, 25].

However, a wide network of MPAs is needed to meet these aspirations when currently such coverage is both uneven and unrepresentative at multiple scales. Assessing the connectivity of marine populations remains a challenge for most species and is even more essential for designing networks of MPAs [26].

Assessing the effectiveness of MPAs is based on comparisons of baseline data prior to the marine reserve establishment against its current state. Then again, without areas closed to human activities/exploitation, it is impossible to evaluate the performance of protective actions, measure the carrying capacity of the system or even imagine it at its virgin unexploited state. A need for long-term scientifically sound monitoring is needed to trace the evolution of species, habitats and processes within MPAs, in comparison to outside protected areas.

Such an endeavour has been undertaken in the recently established Gyaros MPA in the Aegean Sea. The goal was to set a baseline of the Gyaros living marine resources and use the acquired knowledge to assess to assess future status and functioning of the MPA.

Gyaros MPA, although being at its first years of operation, is already showing positive signs (increased species diversity and abundance) suggesting that it is servicing the purpose it was established for. Unfortunately, the other two official MPAs in Greece (National Marine Park of Alonissos, Northern Sporades-NMPANS, established 1992; National Marine Park of Zakynthos–NMPZ established 1999) have fallen short of expectations. NMPANS in the Aegean Sea has been found to be in poor condition and being at the same level as a ‘non-enforced marine protected area and area open to fishing’ [27]. More recently [28], a reduction of catches in the areas adjacent to NMPANS has been observed during the past decade. On the same path, assessment of fish communities in the NMPZ in the Ionian Sea, concluded that existing regulatory scheme falls short of maintaining sufficient protection for the recovery of apex predators or other commercially important fish species [29]. Such under-resourced and poorly funded management bodies and lack of concrete management plans may eventually lead Gyaros MPA down the same road. Some issues that may need the attention of the future management authority are discussed below.

### Fishing period vs spawning period

According to the current legislative scheme governing the Gyaros MPA (Ministerial Decree 389/4.7.2019) specific spatio-temporal access is given to small-scale fishers during a 5-month period: 1st June to 31st October.

Reviewing the spawning period of the species observed in Gyaros MPA from the most recent available sources (Fishbase:—Froese, R. and D. Pauly. Editors. 2021. FishBase. World Wide Web electronic publication. www.fishbase.org, version (02/2021), SeaLifebase: Palomares, M.L.D. and D. Pauly. Editors. 2020. SeaLifeBase. World Wide Web electronic publication. www.sealifebase.org, version (12/2020)), it becomes apparent that for most of the species it coincides with the end of spring—early summer. June in particular is the second most important month with 2/3 of the species exhibiting a peak in spawning (S12 Table in S1 File).

It seems that for an area subject to a special protection regime, like the one herein, such an observation should be taken more seriously into account and should put forward a revision of the fishing period, probably replacing June by another period of the year or at least moving the start of the fishing period by at least 2 weeks (e.g.: from mid-June onwards). Such measures have been applied to conserve Puerto Rican grouper species [30].

### Key species

Top predators shape the structure and functioning of marine communities, playing an important role in sustaining dynamics of food webs [31, 32]. Grouper species (*Epinephelidae*), positioned high in the food chain, are extremely vulnerable to overfishing and in recent decades Mediterranean groupers experienced dramatic population declines [33]. Marine protected areas have been put forward as management tools for protecting their populations inside their boundaries and providing individuals to adjacent fishing areas through the process of spillover and larval export.

Hackradt et al. [34] studied six Mediterranean MPAs to conclude that 5 out of 6 MPAs were able to maintain high abundance, biomass and mean weight of groupers and gave clear indication of spill-over effect. However, they suggest that biomass gradients could only occur where groupers attain sufficient abundance inside MPA limits, indicating a strongly density-dependent process.

In Gyaros MPA, according to our fishing trials, even though groupers’ abundance was higher inside the MPA, a large proportion of them comprised of undersized immature specimens (although larger than the ones residing outside the MPA). A potential reason for the diminished size of key predators may be the extensive recreational fishing (both legal and illegal) that was common practice until 2016 when the surveillance system started (see ‘Threats–Monitoring–Funding’). As a result, any future management scheme should seriously investigate if groupers are to be exploited and if so a temporal restriction during their peak spawning period (summer) may be needed.

### Alien species

In the fishing surveys, invasive alien species comprised an insignificant 1.5% of total catch biomass, however commercial small-scale fishing operations with similar gears outside the MPA did not record such catches. Giakoumi et al. [35], surveying fish and benthic communities in nine Mediterranean MPAs and adjacent unprotected areas, suggested that unprotected sites exhibit lower biomass of invasive fishes compared to MPAs, most likely due to the fishing pressure exerted on alien and native range‐expanding fishes. Other studies advocate in favour of MPAs suggesting that in the context of climate change, protected areas have the potential to build community resilience through a number of mechanisms to promote species and functional stability, and resist the initial stages of tropicalization [36].

It seems that MPA resilience to climate change and alien species colonization may be site specific, depending on a plethora of factors. Nevertheless, warming oceans will inevitably, sooner or later, lead to contraction of resident temperate species and expansion of thermophilous ones [35].

Complementary management actions, such as species‐targeted removals conducted by trained personnel, should be explored as means of effective control of invasive fish populations inside the MPA [37].

### Interaction with Mediterranean monk seals

The initiative for establishing Gyaros island as an MPA originated mainly under the recent findings that it hosts a sizeable Mediterranean monk seal breeding colony, apparently the largest in the Mediterranean Sea [6, 7].

During our fishing trials, encounters with Mediterranean monk seals were frequent with the animals exhibiting no tendency to avoid us; on the contrary they frequently followed our vessel and showed signs of curiosity towards us. However, they have removed significant amounts of fish over night while the net has been casted. It is impossible to estimate the level of these removals, although the fishers suggested that the majority of the catch has been eaten by seals. Besides the catch, damages on the net were also induced, adding to the problem.

Such encounters are also very common in the surrounding islands. Karamanlidis et al. [6] estimates that 21% of small-scale fishing trips in Greece are affected by Mediterranean monk seals. Their study shows that fishers do not take mitigation measures, as they generally believe such measures are not an effective solution to the problem. The majority of fishers regards compensation and/or subsidies to purchase new fishing equipment as the best solution. Interestingly, one out of ten fishers does not hesitate to suggest killing marine animals as the most effective solution.

During the preparatory meetings of the Gyaros consortium of stakeholders (http://cycladeslife.gr/wp-content/plugins/download-attachments/includes/download.php?id=13682), which led to the establishment of the MPA, one of the major objections of fishers towards the establishment of the MPA was that it will allow seals to populate and increase the interaction with their fishing activities. To this end, the initial proposal for the protection zones around the MPA discussed by the Gyaros consortium of stakeholders, included a No Take Zone (NTZ) with the intention to protect Mediterranean monk seals; this area is characterized by numerous marine caves suitable for hosting Mediterranean monk seals (Zone Z1a in S0 Fig in S1 File). Nevertheless, this proposal never materialized in the final agreement.

The need to handle fishers-seals interactions shall be a top priority of management. Reviewing the protection zoning and including a NTZ will have to be revisited and discussed thoroughly. Finally, any compensating scheme to cover damages by seals will require a funding mechanism currently not available.

### Threats–monitoring—funding

Illegal fishing by professional or recreational fishers is always an issue, although the situation has significantly improved recently [38]. Successful monitoring, control and surveillance of activities has been achieved through an elaborate system including:

- a state-of-the-art radar on the mountaintop of Gyaros
- HD cameras
- unmanned aerial vehicles (UAVs)–drones
- WWF crew patrolling the MPA
- collaboration with the Hellenic Coast guard

However, all these have become possible through several projects’ funds that have been completed or heading for conclusion. Currently, a new project is being implemented to pass the function and regular operation of the surveillance system to the Hellenic Coast Guard and to the Natural Environment and Climate Change Agency (NECCA), the new agency responsible for the management of all Greek MPAs.

Assessing if the MPA is functioning and evaluating if it is actually achieving its goals and aspirations, can be attained only by studies like the one herein. The need for continuous periodical monitoring is a must and the future of the MPA depends largely on providing the means for conducting both scientific monitoring as well as enforcing the rules through rigorous surveillance.

In a recent workshop on the “*Post-2020 Regional Strategy for MPAs in the Mediterranean*” (April 8–9 –online UNEP-MEDPAN-SPA/RAC) it has been recognized that most Mediterranean MPAs lack sufficient funds, staff capacity and management plans. Some interesting funding schemes have been put forward e.g.: the MedFund. Three main types of funding were communicated:

Endowment Fund: The capital raised is invested over the long term and investment revenues are used to finance field activities. The capital is preserved.

Sinking Fund: The capital raised and investment revenues are dedicated to directly finance field activities over a period of 5 to 10 years. The capital is consumed.

Revolving Fund: Regular annual revenues are used to finance field activities.

A quite interesting application of a revolving fund in a Moroccan MPA (Al Hoceima National Park), is based on earnings from the fishers. The fee for each fisher is proportional to the amount of catches realized and this has been agreed with the local fishers’ associations (https://blueseeds.org/en/guide-financing-mechanisms/).

If this scheme could be applicable in Gyaros, then the fishers will not only support the funding mechanism but also take over monitoring and surveillance, becoming guardians of the MPA in close collaboration with all key stakeholders (research institutions, local authorities, NGOs, area users).

## Supporting information

S1 Data (PDF)

S1 File (PDF)
