# Supplementary material for: The Gyaros island marine reserve: A biodiversity hotspot in the eastern Mediterranean Sea
Source: PLoS One. 2022 Feb 3;17(2):e0262943. doi: 10.1371/journal.pone.0262943 (PMC8812966; doi:10.1371/journal.pone.0262943)
Supplement: S1 Data — (PDF) [file pone.0262943.s001.pdf]

NAME_SCIENCE	Lmax	AgeMax	Lmat	Sex ratio (M:F)	Spawning period	habitat	feeding
Anthias anthias	27 cm [FAO Zone 37 - MED] (Bauchot, 1987).			Protogynous hermaphrodites (Pickell & Siagian, 2012)		It is found in rocks, gravels, submarine caves on continental shelf and upper slope to about 200 m (Tortonese, 1986)	
Apogonichthyoides nigripinnis	.			Lack evident sexual dimorphism (Vagelli, 2011)	Mouthbrooders, the males incubate the eggs (Golani, et al., 2002)	Adults occur inshore (Paxton, et al., 1989) and in deep offshore reefs (Lieske & Myers, 1994). Found among weeds (Smith & Smith, 1963). Nocturnal species.	Nocturnal species. Feed at night on zooplankton (Golani, et al., 2002)
Aulopus filamentosus						In temperate and sub-tropical waters at depths of up to 1, 000 m but mostly at shallower waters (Souissi, et al., 2010). In coral forests (D'Onghia, et al., 2010) and also in bioturbated fine-grained sedimen on seabed (D'Onghia, et al., 2011).	Mainly fishes, while Crustaceans was secondary/accidental prey [Tunisia] (Romdhani, et al., 2015) also cephalopods was important prey [tyrrhenian sea] (Costa, 1991)
Boops boops	40 cm (Crec'hriou, et al., 2012)	17.8 years (Allam, S. M. 2003)	13.8 cm (M: 14.2 / F: 13.1) (Bottari, et al., 2014)	1:1 (Bottari, et al., 2014)	early January to early May (peak January and February) (El-Agamy, et. al., 2004)<br>February to April (peak in March) (Bottari, et al., 2014)	shelf or coastal pelagic on various bottoms (sand, mud, rocks and seaweeds). Gregarious, ascending to the surface mainly at night. (Frimodt, 1995)	Feeding mainly on crustaceans, also planktophagous. (El-Maremie & El-Mor, 2015)
Chelon labrosus	70 cm (Arechavala-Lopez, et al., 2010)	6 years (Reis & Ateş, 2020)	31 cm (Farrugio & Quignard, 1973)	1.72:1 (Reis & Ateş, 2020)	ber to February (Tsikliras, et al., 2010)	Inshore in schools, frequently entering brackish lagoons and freshwater (Billard, 1997)	Feeds on planktonic and benthonic organisms, mostly oligochaetes, larvae of chironomids and the copepod Euterpina acutifrons. Planktonic forms (34 % of the total prey) were mainly rotifers [Tyrrhenian Sea] (Tosi & Torricelli 1988)
Dactylopterus volitans	48.6 cm (Morey, et al., 2003)					Found on sand, mud or over rocks in sandy areas (Quigley, et al., 2004)	Carnivorous [Ionian sea][Sicily] (Signa, et al., 2017)
Dasyatis pastinaca	114.0 cm TL [Aegean Sea] (Yigin & Ismen, 2012)	16 years [Aegean Sea] (Yigin & Ismen, 2012)	62.5 cm [Aegean Sea] (Yigin & Ismen, 2012)	1:1 [Ionian sea] (Sicily) (Tiralongo, et al., 2020).	Mating season occurred throughout July [Tunisia] (Saadaoui, et al., 2015)	It is found in sandy-muddy substratum mostly at depths less 60 m (McEachran & Capapé, 1984).	Is a generalist carnivorous, mainly feeding on small crustaceans and polychaetes [Ionian sea] (Sicily) (Tiralongo, et al., 2020).
Dentex dentex	100 cm (Baudouin et al., 2016)	36 years (Baudouin et al., 2016)	43.31 cm (M: 52.02 / F: 34.60) Balearic Islands (Morales-Nin and Moranta 1997)<br>22.95 cm (M: 23.32 / F: 22.58) Tunisian coast (Chemmam-Abdelkader 2004).	1:1 (Seasonal variations in the sex ratio) Chemmam-Abdelkader 2004	Spawning period occurs from the end of March until June (Morales-Nin & Moranta, 1997)	demersal fish found from 0 to 200 m depth, living on various substrates such as Posidonia oceanica meadows, rocky bottom with P. oceanica patches, coastal detritic areas, ripples of coarse sand, sandy habitats with Caulerpa and Cymodocea and in the coralligenous community (Marengo et al., 2014)	In the Balearic Islands, the adults feed mainly on fish from the coastal zone (74 %) and on cephalopods (26 %) as secondary prey. The smaller individuals had species from the Posidonia meadow in their stomachs. On the Tunisian coast the common dentex feeds preferentially on fish (84 %), crustaceans (9 %), cephalopods (5 %) and plant remains (2 %) (Chemmam-Abdelkader 2004).
Diplodus annularis	27.5 cm (Crec'hriou,et al., 2012)	17 years (Darmanin, et al., 2019)	M: 10.2 cm / F: 9.7 cm (Mouine et al., 2012)<br>M: 10.5 + - 0.2 cm / F: 10.6 + - 0.3 cm (Chaouch et al., 2013)	(in the eastern Adriatic) is 1:1.12 (Matić-Skoko, 2003)<br>1:2.24 (Gulf of Tunis) (Mouine et al., 2012)	February to July) (Mouine et al., 2012)	Inhabit chiefly Zostera seagrass beds (Darilmaz, 2010) also found on Posidonia beds and sandy bottoms, rarely on rocky bottoms, from 0 to 3 m (Atlantic and northern Mediterranean) or from 0 to 90 m (southern Mediterranean) (Dulčić, et al., 2005).	Feeding on worms, crustaceans, molluscs, echinoderms and hydrozoans (Jardas, 1996). It feeds on macrobenthic algae, bivalves, marine phanerogames, Anthozoa, Polychaeta and Crustacea (juveniles), while adults on mollusca, decapoda, bivalvia, green algae and fish eggs (Matić-Skoko, 2003). The diet of D. vulgaris consists mainly of Crustacea, Annelida, and Mollusca. Algae species were found in lower rates in the stomach contents. Copepods were the most important prey item of D. vulgaris. In addition, Amphipoda, polychaete, and oligochaete species were also included in their stomach contents. Horta et al. (2004) reported that juvenile individuals of D. vulgaris generally feed on Amphipoda, mysids, and algae. Dobroslavić et al. (2013) observed that the diet of juvenile D. vulgaris was based on bivalve larvae and copepods between March and May. The amount of copepods decreased, whereas the amphipods increased from winter to summer. Food availability is one of the most important parameters affecting feeding habits of species (Wassef & Eisawy, 1985).
Diplodus vulgaris	Maltese Islands 30.7 cm (Darmanin et al., 2019) Saros bay 31.9 cm (Cengiz et al., 2019)	16 + years (Darmanin et al., 2019)	17.5 cm (Bouziani et al., 2018)	Rudimentary hermaphrodite, gonochoristic species with batch-spawning fecundity (Buxton & Garratt, 1989). 1:2.75 (Mouine et al., 2012) 1:1.2 (Bouziani et al., 2018)	ber to February) (Mouine et al., 2012)	Euryhaline species that inhabits infra-littoral rocky bottoms and sometimes sandy bottoms up to depths of 160 m, more commonly less than 50 m (Ventura et al., 2015)	Bivalves representing the most important prey type followed by ophiures and polychaeta. However, amphipods and benthic algae were frequently found in the stomachs. In spite of the low amount in stomachs, other groups like hydroids and sea urchins were also abundant. (Sala & Ballesteros, 1997) In the Adriatic Sea is exclusively a carnivorous fish. According to obtained data, benthic echinoids Echinoscyamus pusillus and Psammechinus microtuberculatus were the most common prey and can therefore are classified as main food for D. vulgaris in Adriatic waters. Decapods and bivalves were secondary in importance, except for fish in the largest size classes, where they are the most important prey. Other prey groups were of minor importance and indicated occasional food (Pallaoro et al., 2006).
Epinephelus costae	140 cm (Göthel, 1992)			Protogynous hermaphrodism (Oh et al., 2013)		Demersal fish species inhabiting sandy, muddy, and rocky bottoms, from the shore down to the depth of 300 m, although it is the most abundant in shallow waters (Bañón et al., 2018)	The main prey was fishes (97.1%) and mollusks (2.9%) were occasionally found inside their stomachs. Comparison of ecological categories of prey indicated that E. costae fed on pelagic species (Spanish mediterranean coasts) (López & Orvay, 2005)
Epinephelus marginatus	150 cm (Göthel, 1992)	61 years (Reñones et al., 2007)	M: 68.5 cm / F: 36.7 cm (SL) (Marino et al.,2001)	Protogynous hermaphrodite, During the reproductive period, were 1 : 3.52 Out of this period,it was 1 : 5.14 (Marino et al.,2001)	June until early September (Marino et al.,2001).	Is a necto-benthic species chiefly associated with coastal rocky bottoms and presenting a clear preference for shelter-rich sites. As for many fishes of the coastal zone, its depth range increases with age, first limited to the shallower waters of the infralittoral zone and afterwards extending across the whole continental shelf (Harmelin & Harmelin-Vivien, 1999). Coastal, demersal species, commonly found over irregular rocky bottoms (Almeida Rodrigues Filho et al.,2009) about 300 metres depth, but occurs in higher abundance to 50 metres (Pollard et al., 2018)	Individuals ranging from 20 cm to 60 cm TL feed equally on crabs and fish, and occasionally on molluscs. Preys of larger dusky grouper (>60 cm TL) are in order of importance cephalopods and fish (Harmelin & Harmelin-Vivien, 1999)
Euthynnus alletteratus	107.2 cm [Tunisia] (Hajjej, et al., 2011) 103 cm FL (Fork length)	8 years (Labidi-Neghli et al., 2019)	42cm (Mohamed et al., 2014) East Med<br>M : 43.4 / F : 50.1 (Saber et al., 2018) West Med	1:2.3 (Mohamed et al., 2014).	The spawning period occurs between June and August (Mohamed et al., 2014)	Euthynnus alletteratus is an epipelagic and neritic fish typically occurring in inshore waters. More coastal than other tuna species. This species lives in schools by size together with other scombrid species, but has a tendency to scatter during certain periods of the year. Usually found in coastal waters with swift currents, near shoals and around the warmer waters of thermal fronts and upwellings. Long vertical distribution (Menezes, 1968)	E. alletteratus, ranging from 26.8 to 50.3 cm total length, in the southern Tyrrhenian Sea, ate mostly teleosts, both adults and at larval or juvenile stage (Falautano et al., 2007). Similar results, confirming the dominance of fish preys and the occasional occurrence of crustaceans in the diet were obtained after analysing the stomach content of specimens from the Aegean and Ionian Seas (Zaboukas et al., 2001). Crustaceans and cephalopods seemed to be scarce or not consumed by small little tunny (<44 cm FL), and were totally absent from the stomachs of large little tunny (>51 cm FL) (Bahou et al.,2007). [West Africa]
Labrus mixtus	40 cm (Jardas 1996)	10 years (Matić-Skoko et al., 2013)	M : 25.2 cm / F : 15.23 cm (Matić-Skoko et al., 2013)	Protogynous hermaphrodite and sex change occurred at 26 cm (Matić-Skoko et al., 2013)	April to June (Matić-Skoko et al., 2013)	Labrus mixtus is found on rocky reefs covered by algae or in seagrass (Posidonia oceanica) meadows, at depths ranging from 15 to 40 m (Matić-Skoko et al., 2013). It can also inhabit coralligenous bottoms at depths of 20-100 m (Gomon & Forsyth 1990).	The diet of L. mixtus consisted of at least 60 different prey taxa belonging to 11 major groups (Crustacea, Gastropoda, Bivalvia, Scaphopoda, Pisces, Echinodermata, Polychaeta, Rotatoria, Bryozoa, Porifera and Algae). Crustacean have the highest index of relative importance (%IRI=68.0) and they are the most frequent prey group (%F=67.1), so they can be regarded as the preferred food. Obligatory carnivorous feeder whose diet in the Adriatic Sea is primarily based on crustaceans and gastropods, but also on small fishes, polychaetes, bivalves and echinoderms. (Matić-Skoko et al., 2013)
Mullus surmuletus	32 cm [Balearic Sea] (Reñones, et al., 1995)	11.75 years [Aegean sea] (Kousteni, et al., 2019)	M : 13.92 cm / F : 15.33 cm [Aegean sea] (Kousteni, et al., 2019)	1:1.78 [Balearic Sea] (Alomar, et al., 2017)	March to July (Kousteni, et al., 2019).	Inhabiting mainly rough substrates (Kousteni, et al., 2019) with the highest abundance between 100–200 m of depth (Tserpes et al., 2002). Mullus surmuletus exhibits a pattern of 'inter-depth' migration related to reproduction, meaning that it recruits in shallower habitats over Posidonia oceanica seagrass beds (Kousteni, et al., 2019)	Feeding on Gastropoda, Polychaeta, Copepoda, Ostracoda, Decapoda, Mysidacea, Cumacea, Tanaidacea, Isopoda, Amphipoda, Insecta (larvae) and fishes [Cretan sea] (Maidanou, et al., 2021)
Muraena helena	121 cm (Adriatic) (Matić-Skoko et al., 2014)	10 years (northern coast of Tunisia) (Sallami et al., 2016). 12 years in the Adriatic Sea (Matić-Skoko et al., 2011)	M : 79 cm / F :76 cm TL (Adriatic) (Matić-Skoko et al., 2011)	1.5:1 (northern Tunisia) (Sallami et al., 2019) 1.12:1 (Sallami et al., 2016).	summer with peak in July (Adriatic Sea) (Matić-Skoko et al., 2011)	Abundant within both rocky and sea grass (Posidonia oceanica) habitat (Burns & Kopecky, 2014). From shallow coastal waters to a depth of 800 m, commonly between 100 and 300 m (Sallami et al., 2014). During the days they hide in holes in the bottom slope lurking for their prey. At night they are more eager to leave their hiding spots for a short time, but only in a close perimeter (Matić-Skoko et al. 2011).	Feds mainly on osteichthyans (%IRI = 98.51), while crustaceans (%IRI = 0.65) and cephalopods (%IRI = 0.66) were secondary preys, and occasionally ingested sea grass (%IRI = 0.16). (Sallami et al., 2014)

Pagellus erythrinus	38.9 cm [Aegean sea] (Vassilopoulou & Papaconstantinou, 1990)	14 years [Aegean sea] (Ayyidiz, et al., 2019) 13 years [Ionian sea] (Papaconstantinou, et al., 1988) 8 years [Adriatic] (Maksan, et al., 2019)	M : 14.2 cm / F : 13.4 cm (Cretan Shelf) (Somarakis & Machias, 2001) Tunisian areas: M : 16.75 cm / F : 15.32 cm (Smida et al., 2014).	protogynous hermaphrodism (Smida et al., 2014)	April and ends in August (Tunisia) (Smida et al., 2014). June and August (Adriatic sea) (Tsikliras et al., 2010)	Is a gregarious demersal species living on rocky and muddy–sandy bottoms, between 20 and 300 m depth (Santos et al., 1995)	Polychaetes had the greater IRI in the Corinthiakos Gulf and the Ionian Sea, brachyurans in the Patraikos Gulf. Brachyurans, Polychaetes and decapods were the most important diet items. Decapods contributed more to the weight of prey than polychaetes, but were found in relatively few stomachs. Fishes and cephalopods occurred only occasionally. (Caragitsou & Papaconstantinou, 1988) polychaeta %IRI 10.68 crustacea %IRI 56.40 Mollusca %IRI 24.97 teleostei %IRI 7.95 [Aegean Sea] (Yapici & Filiz, 2019)
Pagrus pagrus	393 FL (Vassilopoulou & Papaconstantinou, 1992)	13 years (Vassilopoulou & Papaconstantinou, 1992)	31.3 FL (Vassilopoulou & Papaconstantinou, 1992)	protogynous hermaphrodism 1 :2.7 (Aegean sea) (Vassilopoulou & Papaconstantinou, 1992)	Spawnig from March till June [Peak in March and April] (Aegean sea) (Vassilopoulou & Papaconstantinou, 1992)	Occurring at depths from 20 to 150 m (Vassilopoulou & Papaconstantinou, 1992).	Feeding on Sipuncula, Polyplacophora, Polychaeta, Crustacea larvae, Copepoda, Decapoda, Mysidacea, Tanaidacea, Isopoda, Amphipoda and Fish [Cretan sea] (Maidanou, et al., 2021)
Palinurus elephas	500	15	100 (74)				
Phycis blennoides	70.3 cm [Ionian Sea] (Matarresse et al., 1998)	20 years (Cohen, et al., 1990)	24.3 cm [West Algeria] (el Amine Benghali, et al., 2014) M : 24.30 cm / F : 30.39 cm [Central Algeria] (Alioua, et al., 2020)	1.1:1 [West Algeria] (el Amine Benghali, et al., 2014) 2.18:1 [Central Algeria] (Alioua, et al., 2020)	November to January [Ionian Sea] (Matarresse et al., 1998). January to March [Balearic sea] (Rotllant, et al., 2002). September to November [West Algeria] (el Amine Benghali, et al., 2014)	It is usually found on muddy or sand bottoms in depths of 100 - 650 m (Bello & Rizzi, 1988).	Preys heavily on crustaceans, which constituted 94.5 % teleost species represented 3.8 % [Balearic Sea] (Morte, et al., 2002)
Phycis phycis	65 cm (Göthel, 1992)	5 years (Matić-Skoko, et al., 2011)	M : 32.98 cm / F : 30.98 [Adriatic] (Glavić, et al., 2014)	1:1.61 [Adriatic] (Glavić, et al., 2014)	The spawning period is from late October to early January [Peak in November to December] [Adriatic] (Glavić, et al., 2014)	Found on hard and sandy-muddy bottoms near rocks at 100-650 m, but sometimes taken at greater depths (Cohen, et al., 1990). Nocturnal, hiding between rocks during the day (Cohen, et al., 1990)	Piscivorous feeder, consuming mainly nektonic animals. Opportunistic browser and feed on practically any consumable-sized animal available (Papaconstantinou & Caragitsou, 1989)
Raja miraletus	58 cm [Tunisia] (Kadri, et al., 2012)	9 years [Tunisia] (Kadri, et al., 2012).	M : 34.31 cm TL (21.91 cm DW) / F : 41.8 cm TL (27.10 cm DW) [Tunisia] (Kadri, et al., 2012).	1:1.12 (Kadri, et al., 2012)		Found over soft bottoms of the shelf and the uppermost slope (Compagno, et al., 1989). Depth range from 17-300 m, and up to depth of 462 m in the eastern Ionian Sea (Mytilineou, et al., 2005).	Decapods (%IRI 88.2), Teleostei (%IRI 2.6), Cephalopods (%IRI 0.8), Polychaeta (%IRI 0.4) [Adriatic] (Šantić, et al., 2013)
Raja radula	TL = 80 cm (Max DW 56 cm) [Tunisia] (Kadri, et al., 2014)	12 years [Tunisia] (Kadri, et al., 2013)	M : 47.05 cm / F : 56.48 cm [Tunisia] (Kadri, et al., 2013)	1: 1.38 [Tunisia] (Kadri, et al., 2014)	Spawns throughout the year with maximum number of egg-cases laid in late spring and summer (Stehmann & Bürkel, 1984)	It inhabits from coastal water to 350 m (Serena, 2005).	Crustacea (%IRI 88.48), Polychaeta (%IRI 1.93), Algae (%IRI 3.36) [Ionian sea][Sicily] (Consalvo, et al., 2010)
Sarpa salpa	79.6 cm [Adriatic][Croatia] (Cetinić, et al., 2002)	15 years (Pallaoro et al., 2008)	M : 21.4 cm / F : 25.3 cm [Adriatic][Croatia] (Cetinić, et al., 2002)	protandrous hermaphrodites 1:1 (Criscoli, et al., 2006)	Two distinct periods were odserved : March to May and from the end of September to November (Criscoli, et al., 2006)	Shallow waters, where there are rocky bottoms or seagrass beds, such as Posidonia oceanica or Cymodocea nodosa (Criscoli, et al., 2006)	Feeds exclusively in algae (Karachle & Stergiou, 2006) also on diatoms and macrophytes (Havelange et al., 1997)
Sciaena umbra	53.1 cm [Balearic Sea] (Grau, et al., 2009) 49.6 cm [Tunisia] (Chakroun-Marzouk & Ktari, 2003)	21 years (Chauvet, 1991)	M : 20 cm / F : 21 cm [Tunisia] (Chakroun-Marzouk & Ktari, 2003)	1:1.44 (Chakroun-Marzouk & Ktari, 2003)	from March to August [Peak in July and August] [Tunisia] (Chakroun-Marzouk & Ktari, 2003)	Occurs in shallow coastal waters mainly on rocky and sandy bottoms, often entering estuaries and more active at night. Also inhabits caves and reefs (Frimodt, 1995)	Feeds on small fishes and crustaceans (Chao, 1986)
Scomber colias	31.2 (2) 33 (35) 37.9 (39)	XII(39)	34.6 (38)	1:0.84 (3)	April-August	pelagic	necton, zooplancton (60)
Scorpaena notata	15.1 (2) 17 (40) 19.9 (41)	VIII (40) XVI (41)	9.2cm (40)	1:1 (40)	Summer (40) July-October (43)	red algae (40) benthic sedentary/ rocky bottoms inside crevices or sea grass meadows, (41)	necton, benth. crust (60)
Scorpaena porcus	27.3 (2) 40.5 (35)	VII(42) VIII(44,45)	F: 17,5 M: 16,7 (45)	1.34:1 (45)	June-August (44) June-September(45)	among rocks and seagrass beds (44)	feeding mainly on benthic preys such as small fishes (gobies and blennies), crustaceans and other invertebrates (44)
Scorpaena scrofa	39.1 (2) 32.2 (35) 30,2 (46) 40.5 (47)	VI (47)	F: 29 M: 24.9 (73)	1:1.3(47)	July-September (47)	rocky, sandy or muddy bottoms from 20 to 200 m (46)	fishes, crustaceans and mollusks (46)benthophagous specialized species that preyed mainly on teleosts followed by crustaceans (48)
Scyliorhinus canicula	M:488 F:467 (68)		M:396 F:399 (68)	1.06:1 (68)	Kousteni et al., 2010; Kousteni et al., 2017	primarily over sandy, gravely or muddy bottoms at depths of a few metres down to 400 m (Compagno 1984).	generalist predator /carnivore with a preference for Teleostei and Cephalopoda (67)
Scyllarides latus							
Sepia officinalis	49 cm (mantle lenght) (Jereb & Roper, 2006)	2 years (Pierce, et al., 2010)	M : 9 cm / F : 13 cm (ML) (Önsoy & Salman, 2005)	1:1.08 (Önsoy & Salman, 2005)	The whole year with two peaks observed. First in March and the second was in June (Önsoy & Salman, 2005)	A neritic, demersal species found in the subtidal zone to depths of 200 m, generally over sandy-muddy substrates. Undergoes seasonal migrations between inshore waters during spring and summer and medium shelf grounds (about 100 m depth) during autumn and winter. In the Mediterranean, large individuals leave deeper water early in spring to migrate to shallower water (Jereb & Roper, 2006)	Feeds on small molluscs, crabs, shrimps, cephalopods and juvenile demersal fishes (Jereb & Roper, 2006).
Serranus cabrilla	19.8 (2) 25.1 (35)	V (49) VI(51)	9.86 (5) 15.0 F (50) 13.2 (51)	1.26:1 (50)	March-May (50,51)	on rocks, Posidonia beds, sandy and muddy bottoms (49)	carnivorous predator decapod crustaceans and teleosts (52)
Serranus scriba	21.8 (4) 24.1 (35) 32.3(54)	XVI (54)	9.3 (53)	simultaneous hermaphrodite	May-July (53)	seagrass meadows	benth. crust., mollusks, worms (60)
Siganus luridus	24.5(56) 25.5 (57)	VI(56), VII(57)	M: 13,9 F: 14,2 (60)		May-July (63)	Mediterranean population inhabit rocky pools & outcrops (60)	grazer (62), herbivore (55)
Sparisoma cretense	40 (36)	VIII (12)	15,5 (60)	0.243 (f36)	July-September (36)		grazer, algae & small invertebrates (36)
Sphyraena sphyraena	40.5 (4)	VIII (33)	M:26.7 F:27,6 (34)	01:00.9	April- August (34)	pelagic	fish, less often on cephalopods and crustaceans (64)
Sphyraena viridensis	113 (59)	IX (59)	M:59,5 F:62,5cm (59)	female dominance (59)	April-June (59)	pelagic	fish (58)
Spicara flexuosa	20 (6)	V (6)	10,3 (60)	protogynus herm	March-June (66)	neritic zone, commonly over Posidonia beds and on sand or muddy bottoms (65)	zooplankton (60)
Spicara maena	22 (2) 26 (35)	VI (8) VIII (10)	M: 11.51 F:13.12 (6)	1:0.24 (1) 1.41:1(10)	April-June (1)	Posidonia beds and sandy or muddy bottoms (1)	omnivorous manner mainly feeds on crustacean (crab, shrimp, isopods, etc.), mollusks (mussel, gastropods, cephalopods, etc.), and zooplanktons (3,7)
Spondyliosoma cantharus	28.7 (2) 31.6 (13) 39.6 (35)	XII (30)	17.8 (13)	22:1 (13)	January- May (13)	the rocky, sand and seagrass beds P. oceanica (31)	small crustaceans (amphipods), polychaetes, ophiuroids and hydrozoans (32)
Squalus blainville	81 (16)	XXVIII (72)	M: 11,3years F:17years (72)				Cephalopods, crustaceans (71)
Stephanolepis diaspros	26.1 (27)	IV (27)	M: 10.58 F: 7.96 (26)	0.87:1 (26)	July-December (26)	coastal rocky substratum, usually with vegetation (28)	benthic organisms: cmstaceans, foraminifers , bivalves, gastropods, sea urchins , and also phytobenthos (29)
Symphodus tinca	23.7 (35)	XV (59)	10 (60)	protogynon (59)	April-July (59)	near rocks, in eel-grass beds (60)	sea urchins, ophiuroids, bivalves, shrimps & crabs (59)
Torpedo marmorata	M:38,6 F:61,2 (69)		M:25,5 F:35,5 (69)		mate: Nov-Jan ovulate: Dec-Feb birth:December (10-12m gestation) (69)		fish, cephalopods (69)
Trachinus radiatus	50.7 (24) 40.4 (35)	XV (24)	M: 25.2 F:24.3 (25)	1.42:1 (25)	May- September (25)	sand and mud bottoms on the continental shelf (60)	
Trachurus trachurus	37 (11) 28 (35)	X (11)	22 (20)		December- July (20)	SemiPelagic (19)	zooplanktophagous: Polychaeta, Crustacea, Mollusca, Chaetognatha and Osteichthyes (19)
Trigloporus lastoviza	30 (21)	VIII (23)	139 (23)	F:M 1.1:1 (21)	November-June (21)	Found over rocks and sand	Decapoda reptantia, Mysidacea, Cephalopoda and Decapoda natantia (22)
Uranoscopus scaber	30.6 (2) 28.4(35)	V (14)	M:13.75 F:11.76 (18)	0.94:1 (17)	March-September (17)	Benthic sundy-muddy (17)	finfish, benthic invert (60)
Zeus faber	52.8 (15) 55(35)	XVIII (15)	25.4 (15)	1.6:1 (15)	partial spawning of Z. faber continued throughout the year, with increases from January to June and between August to September (15)	Benthic	Pisces & crustaceans (15)

BIBLIO	Citation	Area
1	Cengiz, 2019	N. Aegean
2	Karakulak et al., 2006	N. Aegean
4	Kapiris & Klaoudatos, 2011	C. Aegean (Argolikos)
5	Iikyaz et al., 2018	C. Aegean
6	Soykan et al., 2010	C. Aegean
7	Can, A. & Bilecenoğlu M., 2005. Atlas of Deep Sea Fishes of Turkey (in Turkish). Arkadaş Yayın Evi, Ankara, Turkey, 108pp.	
8	Saygili et al., 2016	N. Aegean
9	Ozvarol et al., 2014	NE Mediterranean (Turkey)
10	Dulcic, 2000	Adriatic
11	Abaunza et al. (2003)	Aegean
12	Papakonstantinou et al., 1998	Aegean
13	Mouinie et al., 2011	Gulf of Tunis
14	Kartas & Bondka (1986	Gulf of Gabes
15	Ismen et al. (2013)	N. Aegean
16	Marouani et al. (2007, 2010)	Gulf of Gabes
17	Coker et al., 2008	Aegean
18	Ak et al., 2011	Black Sea
19	Byahan & Sever, 2009	Aegean
20	Karlou Riga & Economides, 1997	Aegean
21	Papakonstantinou et al., 1983	Aegean
22	Terrats et al., 1999	Aegean
23	Papakonstantinou, 1985	Aegean
24	Hamed et al., 2019	Tunis
25	Hamed & Chakroun-Marzouk, 2017	Tunis
26	Rim & Mohamed-Nejmeddine, 2011	Gulf of Gabes
27	El Ganainy & Sabra, 2008	Gulf of Suez
28	Golani et al., 2002	
29	Zouaru Ktali et al., 2008	Gulf of Gabes
30	Boughamou et al., 2015	Algerie
31	Guidetti, 2000	
32	Dulcic, 2006	Adriatic
33	Allam et al., 2004b	Egypt
34	Allam et al., 2004a	Egypt
35	Moutopoulos & Stergiou, 2000	Aegean
36	de Girolamo et al., 1999	Lampedusa katv apo th sikelia
37	Cengiz, 2012	N. Aegean
38	Cengiz, 2020	N. Aegean
39	Spetsiotis et al., 2016	epsad aegean
40	Ordines et al., 2009	W. Mediterr Balearic Isl.
41	Scarcella et al., 2011	Adriatic
42	Scarcella et al., 2011b	Adriatic
43	Munoz et al., 2005	W. Mediterr
44	La Mesa et al., 2010	Adriatic
45	Bilgin & Celik, 2009	Black Sea
46	Arslan & Bostanci, 2019	Aegean
47	Shahrani & Shakman, 2015	Libya
48	Cabiddu et al., 2010	Sardinia
49	Tserpes & Tsimenides, 2001	Cretan Sea, Aegean
50	Torcu-Koc et al., 2004	NW Aegean
51	Ilhan et al., 2010	Aegean
52	Rachedi et al., 2018	Algerie
53	Zorica et al., 2006	Adriatic
54	Zorica et al., 2010	Adriatic
55	Bariche, 2006	E. Mediterranean
56	Bariche, 2004	E. Mediterranean
57	Shakman et al., 2008	E. Mediterranean
58	Barreiros, J.P.; Santos, R.S.; Borba, A.E. (2002). "Food habits, schooling and predatory behaviour of the Yellowmouth Barracuda, Sphyraena viridensis Cuvier, 1829 (Perciformes: Sphyraenidae) in the Azores". «Cybium –International Journal of Ichthyology», 26(2): 83-88. ISSNe 2101-0315.	N. Atlantic (Azores)

59	Quignard, J.-P. and A. Pras, 1986. Labridae. p. 919-942. In P.J.P. Whitehead, M.-L. Bauchot, J.-C. Hureau, J. Nielsen and E. Tortonese (eds.) Fishes of the north-eastern Atlantic and the Mediterranean. UNESCO, Paris. Vol. 2.	
60	FishBase	
61	Pinnegar, J.K. and N.V. Polunin, 2000. Contributions of stable-isotope data to elucidating food webs of Mediterranean rocky littoral fishes. Oecologia 122:399-409.	
62	Stergiou, K.I., 1988. Feeding habits of the Lessepsian migrant Siganus luridus in the eastern Mediterranean, its new environment. J. Fish Biol. 33(4):531-543.	
63	Bariche, M., M. Harmelin-Vivien and J.-P. Quignard, 2003. Reproductive cycles and spawning periods of two Lessepsian siganid fishes on the Lebanese coast. J. Fish Biol. 62(1):129-142.	
64	Ben-Tuvia, A., 1986. Sphyraenidae. p. 1194-1196. In P.J.P. Whitehead, M.-L. Bauchot, J.-C. Hureau, J. Nielsen and E. Tortonese (eds.) Fishes of the north-eastern Atlantic and the Mediterranean. UNESCO, Paris. Vol. 3.	
65	Tortonese, E., 1986. Centracanthidae. p. 908-911. In P.J.P. Whitehead, M.-L. Bauchot, J.-C. Hureau, J. Nielsen and E. Tortonese (eds.) Fishes of the north-eastern Atlantic and the Mediterranean. UNESCO, Paris. vol. 2.	
66	Soykan, O., A.T. Ilkyaz, G. Metin and H.T. Kinacigil, 2010. Growth and reproduction of blotched picarel (Spicara maena Linnaeus, 1758) in the central Aegean Sea, Turkey. Turk. J. Zool. 34:453-459.	
67	Kousteni V., P. K. Karachle and P. Megalofonou, 2017. Diet of the small-spotted catshark Scyliorhinus canicula in the Aegean Sea (eastern Mediterranean). Marine Biology Research, 13: 161-173.	
68	Kousteni V. , M. Kontopoulou & P. Megalofonou (2010) Sexual maturity and fecundity of Scyliorhinus canicula (Linnaeus, 1758) in the Aegean Sea, Marine Biology Research, 6:4, 390-398.	
69	Abdel-Aziz SH (1994) Observations on the biology of the common torpedo (*Torpedo torpedo* , Linnaeus, 1758) and marbled electric ray (*Torpedo marmorata* , Risso, 1810) from Egyptian Mediterranean waters. *Marine and Freshwater Research* **45**, 693-704.	
70	Jacobsen IP, Bennett MB (2013) A Comparative Analysis of Feeding and Trophic Level Ecology in Stingrays (Rajiformes; Myliobatoidei) and Electric Rays (Rajiformes: Torpedinoidei). PLoS ONE 8(8): e71348.	
71	Kousteni et al., 2017b	
72	Kousteni & Megalofonou, 2015	
73	Matić-Skoko, Sanja, et al. "The biological traits of the large red scorpionfish, Scorpaena scrofa: Temporal and ontogenetic dynamics." *Estuarine, Coastal and Shelf Science* 152 (2015): 91-99.	
74	Goñi, R., A. Quetglas and O. Reñones 2003 Size at maturity, fecundity and reproductive potential of a protected population of the spiny lobster Palinurus elephas (Fabricius, 1787) from the western Mediterranean. Mar. Biol. 143(3):583-592.	W. Mediterranean (Spain)
