# Supplementary material for: The Gyaros island marine reserve: A biodiversity hotspot in the eastern Mediterranean Sea
Source: PLoS One. 2022 Feb 3;17(2):e0262943. doi: 10.1371/journal.pone.0262943 (PMC8812966; doi:10.1371/journal.pone.0262943)
Supplement: S1 File — (PDF) [file pone.0262943.s002.pdf]

Supplementary material

Table S0. Hauls of commercial SSF vessels outside the MPA monitored by on-board observers implementing EU DCF.

Haul	Date	Season	Area	Mesh size	Net length	Depth (m)
1	7/2/2018	winter	Siros	32	745	54
2	8/2/2018	winter	Siros	32	670	54
3	12/4/2018	spring	South Syros	32	1560	16
4	14/4/2018	spring	South Syros	30	450	39
5	23/5/2018	spring	Siros	32	1100	40
6	7/6/2018	summer	Siros	32	760	54
7	13/8/2018	summer	South Syros	30	430	6
8	4/10/2018	autumn	South Syros	32	1200	49
9	8/10/2018	autumn	South Syros	32	414	87
10	12/1/2019	winter	South Syros	30	430	59
11	10/2/2019	winter	South Syros	32	1380	49
12	12/2/2019	winter	East Syros	30	2630	49
13	3/8/2019	summer	South Syros	34	3300	76

Table S1. Catch composition in weight by sampling location for the experimental fishing trials with nets in Gyaros island

Species	St1	St2	St3	St4	St5	Total
Sparisoma cretense	**51.9%**	**49.8%**	0.0%	0.0%	0.0%	**32.1%**
Scorpaena scrofa	10.4%	13.9%	9.5%	14.0%	0.0%	10.8%
Palinurus elephas	0.0%	0.0%	**27.0%**	**29.8%**	0.0%	7.3%
Pagrus pagrus	0.4%	0.9%	9.5%	5.5%	**22.5%**	4.9%
Euthynnus alletteratus	10.1%	1.6%	0.0%	0.0%	0.0%	3.6%
Muraena helena	8.0%	2.8%	0.0%	0.0%	0.0%	3.3%
Scyliorhinus canicula	0.0%	0.0%	15.4%	8.7%	0.0%	3.1%
Phycis phycis	0.4%	1.0%	3.1%	5.1%	12.5%	2.9%
Mullus surmuletus	1.4%	2.0%	8.3%	1.8%	4.2%	2.8%
Sepia officinalis	3.5%	1.4%	0.9%	6.5%	1.0%	2.6%
Others	13.8%	26.7%	26.3%	28.7%	**59.7%**	26.7%
Total species/taxa identified	**31**	**40**	**15**	**23**	**30**	**75**

Table S2. Biomass (in kg/1000m of trammel net) by sampling location for the experimental fishing trials with nets in Gyaros island

Species	St1	St2	St3	St4	St5	Total
Sparisoma cretense	**8.03**	**8.94**	0.00	0.00	0.00	**3.49**
Scorpaena scrofa	1.61	2.49	0.63	1.06	0.00	1.17
Palinurus elephas	0.00	0.00	**1.79**	**2.24**	0.00	0.80
Pagrus pagrus	0.06	0.17	0.63	0.42	**1.45**	0.53
Euthynnus alletteratus	1.56	0.28	0.00	0.00	0.00	0.39
Muraena helena	1.23	0.49	0.00	0.00	0.00	0.36
Scyliorhinus canicula	0.00	0.00	1.02	0.65	0.00	0.34
Phycis phycis	0.06	0.17	0.20	0.38	0.81	0.32
Mullus surmuletus	0.22	0.35	0.55	0.13	0.27	0.31
Sepia officinalis	0.54	0.25	0.06	0.49	0.07	0.28
Others	2.14	4.78	1.75	2.16	**3.84**	2.96
Total	**15.45**	**17.93**	**6.64**	**7.52**	**6.44**	**10.95**

Table S3. Results of an ANOVA test on the effects of Year and Station on biomass

Factors on CPUEW	Df	Sum Sq	Mean Sq	F value	Pr(>F)
YEAR	2	140.8	70.41	0.767	0.4727
STATION	4	1131.2	282.8	3.079	0.0293
Residuals	33	3031.1	91.85		

Table S4. Results of an ANOVA test ANOVA test, with substrate, year, season and depth as driving factors of biomass for four main species

Analysis of Variance for *Scyliorhinus.canicula*

Source	*Sum of Squares*	*Df*	*Mean Square*	*F-Ratio*	*P-Value*
Model	6.80685	12	0.567237	1.68	0.1274
Residual	9.10635	27	0.337272		
Total (Corr.)	15.9132	39			

Type III Sums of Squares

Source	*Sum of Squares*	*Df*	*Mean Square*	*F-Ratio*	*P-Value*
**substrate**	5.61614	7	0.802306	2.38	**0.0495**
Year	0.771635	2	0.385817	1.14	0.3335
Season	1.01184	3	0.337281	1.00	0.4079
Residual	9.10635	27	0.337272		
Total (corrected)	15.9132	39			

Analysis of Variance for *Scorpaena.scrofa*

Source	*Sum of Squares*	*Df*	*Mean Square*	*F-Ratio*	*P-Value*
Model	49.0002	12	4.08335	2.78	0.0133
Residual	39.6426	27	1.46824		
Total (Corr.)	88.6428	39			

Type III Sums of Squares

Source	*Sum of Squares*	*Df*	*Mean Square*	*F-Ratio*	*P-Value*
**substrate**	34.9913	7	4.99876	3.40	**0.0098**
Year	4.56869	2	2.28434	1.56	0.2293
Season	11.621	3	3.87367	2.64	0.0699
Residual	39.6426	27	1.46824		
Total (corrected)	88.6428	39			

Analysis of Variance for *Sparisoma.cretense*

Source	*Sum of Squares*	*Df*	*Mean Square*	*F-Ratio*	*P-Value*
Model	1237.41	12	103.117	3.78	0.0020
Residual	735.608	27	27.2447		
Total (Corr.)	1973.01	39			

Type III Sums of Squares

Source	*Sum of Squares*	*Df*	*Mean Square*	*F-Ratio*	*P-Value*
**substrate**	933.059	7	133.294	4.89	**0.0012**
Year	39.8619	2	19.9309	0.73	0.4905
Season	42.1403	3	14.0468	0.52	0.6751
Residual	735.608	27	27.2447		
Total (corrected)	1973.01	39			

Analysis of Variance for *Mullus.surmuletus*

Source	*Sum of Squares*	*Df*	*Mean Square*	*F-Ratio*	*P-Value*
Model	4.37223	12	0.364353	1.27	0.2911
Residual	7.74871	27	0.286989		
Total (Corr.)	12.1209	39			

Type III Sums of Squares

Source	*Sum of Squares*	*Df*	*Mean Square*	*F-Ratio*	*P-Value*
substrate	0.780012	7	0.11143	0.39	0.9010
**Year**	1.46109	2	0.730545	2.55	**0.0471**
Season	1.14731	3	0.382435	1.33	0.2844
Residual	7.74871	27	0.286989		
Total (corrected)	12.1209	39			

Table S5. SIMPER Analysis: Average dissimilarity (%) of species abundance per sampling site

Sampling area	St 5-Fouis	St 4-Colata	St 3-Fournaki	St 2-Glaronissi
**St 1-Fyllada**	89.69%	89.25%	89.28%	65.91%
**St 2-Glaronissi**	87.57%	88.98%	88.90%	
**St 3-Fournaki**	90.95%	68.22%		
**St 4-Colata**	91.23%			

Table S6. SIMPER Analysis results for the species that are responsible for the dissimilarities among the 5 sampling sites examined in Gyaros MPA. Only the species with a contribution over 5% in the dissimilarity are presented.

**St1.Fyllada – St2.Glaronissi**	**St1.Fyllada – St3.Fournaki**	**St1.Fyllada – St4.Colata**	**St1.Fyllada – St.5.Fouis**
S.cretense **10.54%**	*S. cretense* 12.52%	*S. cretense* 13.24%	*S. cretense* 13.29%
D. calidus **6.79%**	*S. canicula* 7.53%	*S. scrofa* 7.02%	*S. scrofa* 8.93%
S. scrofa **5.84%**	*S. scrofa* 6.53%	*S. canicula* 5.50%	*S.cantharus* 5.34%
S. luridus **5.59%**	*P. erythrinus* 5.04%	*P. elephas* 5.03%	
D. vulgaris **5.27%**			
**St2.Glaronissi – St3.Fournaki**	**St2.Glaronissi – St4.Colata**	**St2.Glaronissi – St5.Fouis**	
S. cretense **9.64%**	*S. cretense* 9.96%	*S. cretense* 10.25%	
S. canicula **6.71%**	*S. scrofa* 6.14%	*S. scrofa* 7.64%	
S. scrofa **5.88%**	*D. calidus* 5.76%	*D. calidus* 5.88%	
D. vulgaris **5.61%**	*D. vulgaris* 5.45%	*D. vulgaris* 5.23%	
D. calidus **5.59%**			
**St3.Fournaki – St4.Colata**	**St3.Fournaki – St5.Fouis**		
S. canicula **11.30%**	*S. canicula* 10.22%		
C. cidaris **10.68%**	*P. erythrinus* 7.23%		
P. elephas **10.21%**	*P. pagrus* 6.89%		
M. surmuletus **8.60%**	*M. surmuletus* 6.50%		
P. erythrinus **8.05%**	*P. elephas* 6.42%		
P. pagrus **7.94%**	*C. cidaris* 6.29%		
S. affinis **7.29%**	*S. cantharus* 6.22%		
S. scrofa **6.61%**	*S. scrofa* 5.19%		
**St4.Colata – St5.Fouis**			
P. elephas **7.32%**			
S. canicula **7.29%**			
S. cantharus **6.42%**			
P. pagrus **6.32%**			
P. ertythrinus **6.11%**			

Table S7. Sex ratios of the most common commercial species observed in Gyaros MPA.

Species	Females	Males
Scorpaena scrofa	84.4%	15.6%
Mullus surmuletus	82.8%	17.2%
Pagrus pagrus	75.0%	25.0%
Pagellus erythrinus	70.8%	29.2%
Scyliorhinus canicula	57.1%	42.9%
Phycis phycis	50.0%	50.0%
Sparisoma cretense	46.0%	54.0%
Palinurus elephas	34.8%	65.2%
Sepia officinalis	34.6%	65.4%
Total	**57.2%**	**42.8%**

Table S8. Catch composition (in weight) of the SSF vessels fishing in the surrounding areas of Gyaros MPA.

Species	% of total catch	Species	% of total catch
Sepia officinalis	11.62%	*Phycis phycis*	0.63%
Raja clavata	10.38%	*Raja radula*	0.60%
Scorpaena scrofa	9.45%	*Scyllarides latus*	0.54%
Palinurus elephas	8.95%	*Dentex dentex*	0.50%
Mustelus mustelus	6.56%	*Diplodus annularis*	0.49%
Sparisoma cretense	4.64%	*Torpedo torpedo*	0.46%
Mullus surmuletus	3.56%	*Trigloporus lastoviza*	0.36%
Lophius budegassa	3.21%	*Diplodus sargus*	0.35%
Scyliorhinus canicula	3.08%	*Serranus cabrilla*	0.33%
Scomber colias	2.93%	*Liza aurata*	0.32%
Diplodus vulgaris	2.70%	*Conger conger*	0.32%
Oblada melanura	2.51%	*Dactylopterus volitans*	0.26%
Muraena helena	2.32%	*Serranus scriba*	0.25%
Pagellus erythrinus	2.23%	*Zeus faber*	0.24%
Scorpaena porcus	2.10%	*Dasyatis pastinaca*	0.22%
Euthynnus alleteratus	1.82%	*Raja miraletus*	0.21%
Dentex macrophthalmus	1.79%	*Sciaena umbra*	0.21%
Uranoscopus scaber	1.69%	*Loligo vulgaris*	0.20%
Pagrus pagrus	1.55%	*Trachurus trachurus*	0.19%
Symphodus tinca	1.43%	*Merluccius merluccius*	0.18%
Sarda sarda	1.19%	*Labrus merula*	0.18%
Synodus saurus	1.12%	*Liocarcinus corrugatus*	0.10%
Maja squinado	1.09%	*Calappa granulata*	0.10%
Spicara maena	1.03%	*Illex coindetii*	0.08%
Spondyliosoma cantharus	0.76%	*Sarpa salpa*	0.07%
Epinephelus costae	0.70%	*Xyrichtys novacula*	0.06%
Auxis rochei	0.67%	*Synapturichthys kleinii*	0.06%
Sphyraena sphyraena	0.67%	*Bothus podas*	0.05%
Boops boops	0.66%	*Pagellus acarne*	0.01%

Table S9. Length statistics for the species caught by SSF vessels fishing in the surrounding areas of Gyaros MPA. Species ordered by sample size. Results can be considered meaningful only for the species having a sample size >= 10.

Species	Mean	Min	Max	Count	MLS	% below MLS
Sepia officinalis	128.5	94	183	61		
Scorpaena scrofa	219.1	153	385	57		
Dentex macrophthalmus	163.6	136	200	42		
Mullus surmuletus	215.3	151	297	39	110	0
Scorpaena porcus	165.9	120	239	33		
Diplodus vulgaris	196.1	152	267	28	180	14
Diplodus annularis	126.6	102	157	25	120	32
Scyliorhinus canicula	389.8	317	458	25		
Sparisoma cretense	261.6	208	302	24		
Spicara maena	172.2	150	197	20		
Uranoscopus scaber	192.1	135	322	16		
Oblada melanura	263.6	226	289	15		
Palinurus elephas	107.3	82	119	15	90	7
Scomber colias	313.5	202	380	15	180	0
Synodus saurus	253.5	202	311	14		
Symphodus tinca	213.6	168	286	12		
Raja clavata	656.4	583	738	10		
Boops boops	193.4	100	240	9	100	0
Liocarcinus corrugatus	38.1	34	44	8		
Pagellus erythrinus	287.3	205	316	7	150	0
Serranus cabrilla	184.7	162	205	7		
Pagrus pagrus	230.1	125	348	7		
Euthynnus alleteratus	357.4	341	373	5		
Muraena helena	687.4	556	877	5		
Mustelus mustelus	805.2	627	1045	5		
Phycis phycis	266.2	177	309	5		
Serranus scriba	180.6	168	188	5		
Trigloporus lastoviza	224.4	205	262	5		
Spondyliosoma cantharus	255	191	328	4		
Dentex dentex	266	217	292	3		
Sciaena umbra	216.7	198	230	3		
Bothus podas	152.5	135	170	2		
Calappa granulata	57.5	47	68	2		
Conger conger	492	290	694	2		
Diplodus sargus	185	179	191	2	230	100
Epinephelus costae	353.5	287	420	2	450	100
Pagellus acarne	100	99	101	2		
Raja miraletus	469.5	468	471	2		
Trachurus trachurus	270.5	235	306	2	150	0
Zeus faber	208	177	239	2		
Auxis rochei	412	412	412	1		
Dactylopterus volitans	364	364	364	1		
Dasyatis pastinaca	400	400	400	1		
Illex coindetii	168	168	168	1		
Labrus merula	281	281	281	1		
Liza aurata	377	377	377	1		
Loligo vulgaris	180	180	180	1		
Lophius budegassa	732	732	732	1		
Maja squinado	182	182	182	1		
Merluccius merluccius	353	353	353	1	200	0
Raja radula	515	515	515	1		
Sarda sarda	536	536	536	1		
Sarpa salpa	211	211	211	1		
Scyllarides latus	118	118	118	1		
Synapturichthys kleinii	224	224	224	1		
Sphyraena sphyraena	688	688	688	1		
Torpedo torpedo	328	328	328	1		
Xyrichtys novacula	196	196	196	1		

Table S10. Catch composition (in weight) and biomass (kg/1000 m of trammel net) from the logbooks of the same fishing vessel used for the experimental fishing trials in Gyaros MPA during 2017-2018 in the islands surrounding Gyaros MPA.

Species	Catch composition	CPUEW (kg/1000 m of net)
Scorpaena scrofa	29.8%	0.97
Sparisoma cretense	16.8%	0.55
Oblada melanura	13.8%	0.45
Spicara maena	11.6%	0.38
Euthynnus alletteratus	6.3%	0.21
Boops boops	5.5%	0.18
Scomber colias	3.3%	0.11
Sepia officinalis	3.3%	0.11
Mullus surmuletus	2.8%	0.09
Palinurus elephas	2.8%	0.09
Epinephelus costae	1.7%	0.05
Zeus faber	0.8%	0.03
Phycis spp.	0.8%	0.03
Scyllarides latus	0.7%	0.02
**Total**	**100.0%**	**3.26**

Table S11. Average length (in mm) of species captured in and out of Gyaros MPA.

Species	In	Out	% difference In-Out
Sciaena umbra	352.7	216.7	63%
Sarpa salpa	306.5	211.0	45%
Dentex dentex	372.0	266.0	40%
Pagrus pagrus	312.2	230.1	36%
Phycis phycis	348.1	266.2	31%
Uranoscopus scaber	244.5	192.1	27%
Muraena helena	874.0	687.4	27%
Scorpaena porcus	207.5	165.9	25%
Mullus surmuletus	269.0	215.3	25%
Dasyatis pastinaca	490.0	400.0	23%
Scorpaena scrofa	265.4	219.1	21%
Labrus merula	340.0	281.0	21%
Diplodus annularis	152.3	126.6	20%
Palinurus elephas	127.8	107.3	19%
Serranus scriba	210.9	180.6	17%
Sepia officinalis	144.8	128.5	13%
Sparisoma cretense	291.7	261.6	11%
Trachurus trachurus	298.0	270.5	10%
Trigloporus lastoviza	247.0	224.4	10%
Scyliorhinus canicula	414.2	389.8	6%
Epinephelus costae	374.8	353.5	6%
Oblada melanura	278.3	263.6	6%
Symphodus tinca	219.0	213.6	3%
Scomber colias	317.3	313.5	1%
Spicara maena	172.0	172.2	0%
Diplodus vulgaris	195.0	196.1	-1%
Pagellus erythrinus	281.2	287.3	-2%
Zeus faber	200.0	208.0	-4%
Dactylopterus volitans	338.0	364.0	-7%
Raja clavata	607.0	656.4	-8%
Spondyliosoma cantharus	234.9	255.0	-8%
Raja miraletus	410.7	469.5	-13%
Scyllarides latus	95.3	118.0	-19%
Raja radula	415.7	515.0	-19%
Serranus cabrilla	142.7	184.7	-23%
Sphyraena sphyraena	523.5	688.0	-24%

Table S12. Spawning period chart for the species of commercial interest in Gyaros MPA. Fishing period inside the MPA is highlighted by a red box.

Species	Spawning period											
	Jan	Feb	Mar	Apr	May	Jun	Jul	Aug	Sep	Oct	Nov	Dec
Boops boops	x	x	x	x								
Chelon labrosus		x	x	x	x	x						
Dentex dentex				x	x	x						
Diplodus annularis		x	x	x								
Diplodus puntazzo									x			
Diplodus vulgaris	x											x
Epinephelus costae						x	x	x	x			
Epinephelus marginatus						x	x	x	x			
Euthynnus alletteratus					x	x	x	x				
Labrus merula		x	x	x	x							
Labrus mixtus			x	x	x	x						
Mullus surmuletus					x	x	x					
Oblada melanura				x	x	x						
Pagellus erythrinus			x	x	x	x	x	x	x	x	x	
Pagrus pagrus				x	x	x	x	x	x			
Palinurus elephas	x	x							x	x	x	x
Phycis blennoides	x	x	x	x	x							
Phycis phycis	x	x	x	x	x							
Pseudocaranx dentex						x	x	x				
Raja clavata		x	x	x	x	x	x	x	x			
Raja miraletus	x	x	x	x	x	x	x	x	x	x	x	x
Raja polystigma	x	x	x	x	x	x	x	x	x	x	x	x
Raja radula	x	x	x	x	x	x	x	x	x	x	x	x
Sarpa salpa			x	x	x				x	x	x	
Sciaena umbra			x	x	x	x	x	x				
Scomber colias					x	x	x					
Scorpaena notata									x			
Scorpaena porcus			x	x	x	x	x	x	x			
Scorpaena scrofa							x	x	x			
Scyliorhinus canicula	x	x	x	x	x	x	x				x	x
Scyllarides latus						x	x	x				
Sepia officinalis			x	x	x							
Serranus cabrilla				x	x	x	x					
Serranus scriba				x	x	x	x	x				
Siganus luridus					x	x	x					
Sparisoma cretense								x	x	x		
Sphyraena sphyraena						x						
Sphyraena viridensis						x						
Spicara flexuosa			x	x	x	x						
Spicara maena			x	x	x	x						
Spondyliosoma cantharus			x	x	x							
Squalus acanthias	x	x	x	x	x	x	x	x	x	x	x	x
Squalus blainville	x	x	x	x	x	x	x	x	x	x	x	x
Symphodus tinca				x	x	x	x					
Trachinus radiatus			x	x	x	x	x	x				
Trachurus trachurus				x	x	x	x	x	x	x		
Trigloporus lastoviza	x	x	x	x								
Uranoscopus scaber				x	x	x	x	x				
Zeus faber			x	x	x				x	x	x	
**Number of species**	**12**	**15**	**25**	**33**	**34**	**33**	**26**	**21**	**19**	**11**	**10**	**8**
**% spawning**	24%	31%	51%	67%	69%	67%	53%	43%	39%	22%	20%	16%

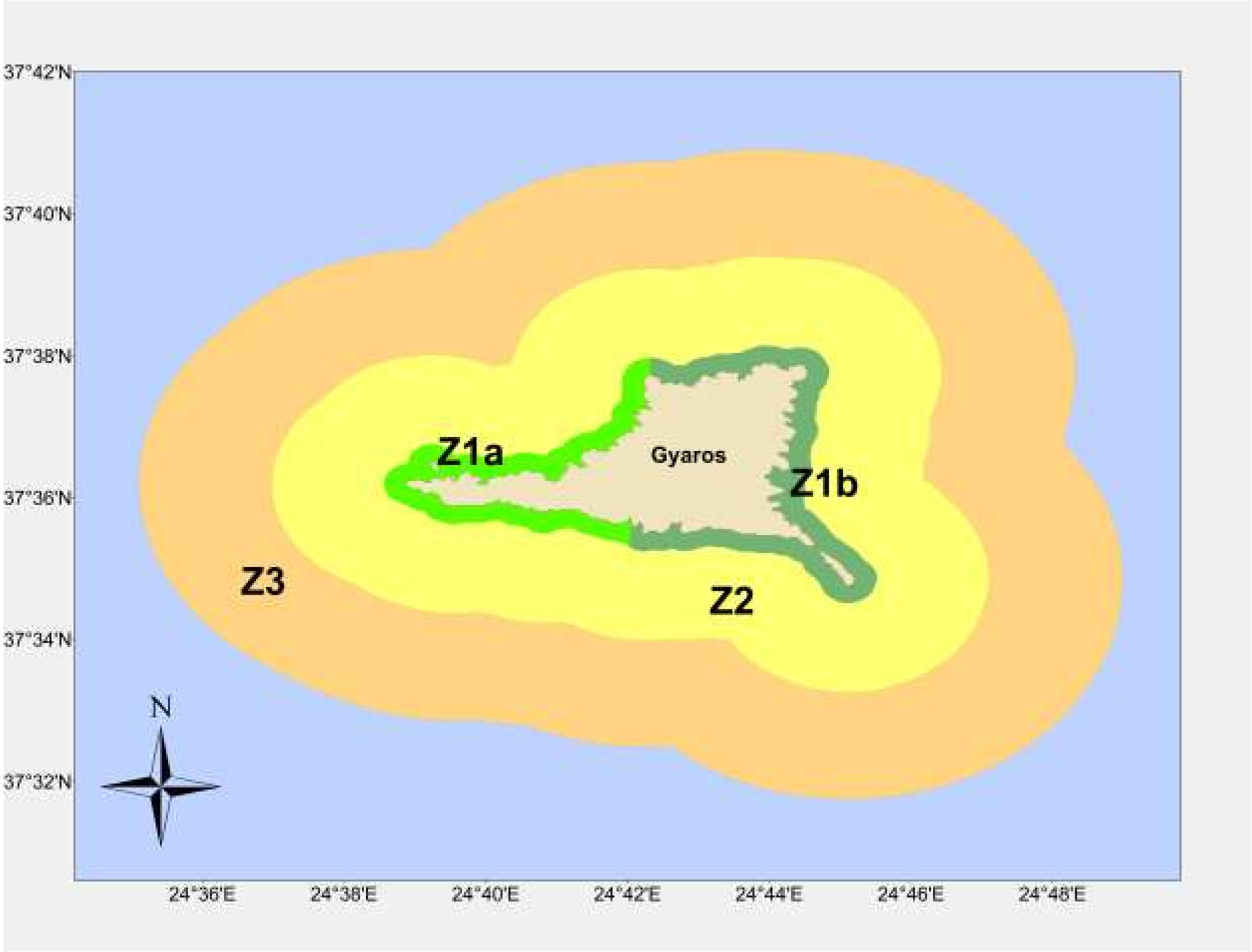

Fig. S0. Map depicting the protection zoning around Gyaros island
(protection zones Z1a: light green, Z1b: dark green, Z2: yellow, Z3: orange).
Exploitation is allowed from 1st of June till end of October, by both static nets and long-lines in zones Z1 and Z3, while only bottom static long-lines are allowed in zone Z2. Zone Z2 is largely dominated by coralligenous substrates which according to national legislation (FEK 3937/A' 60/ 31.03.2011) fishing with any type of towed gear or static nets is prohibited.
(Greece basemap reprinted from Kavadas et al., 2012 - https://doi.org/10.12681/mms.324 under a CC BY license, with permission from Stefanos Kavadas-HCMR, original copyright 2004)

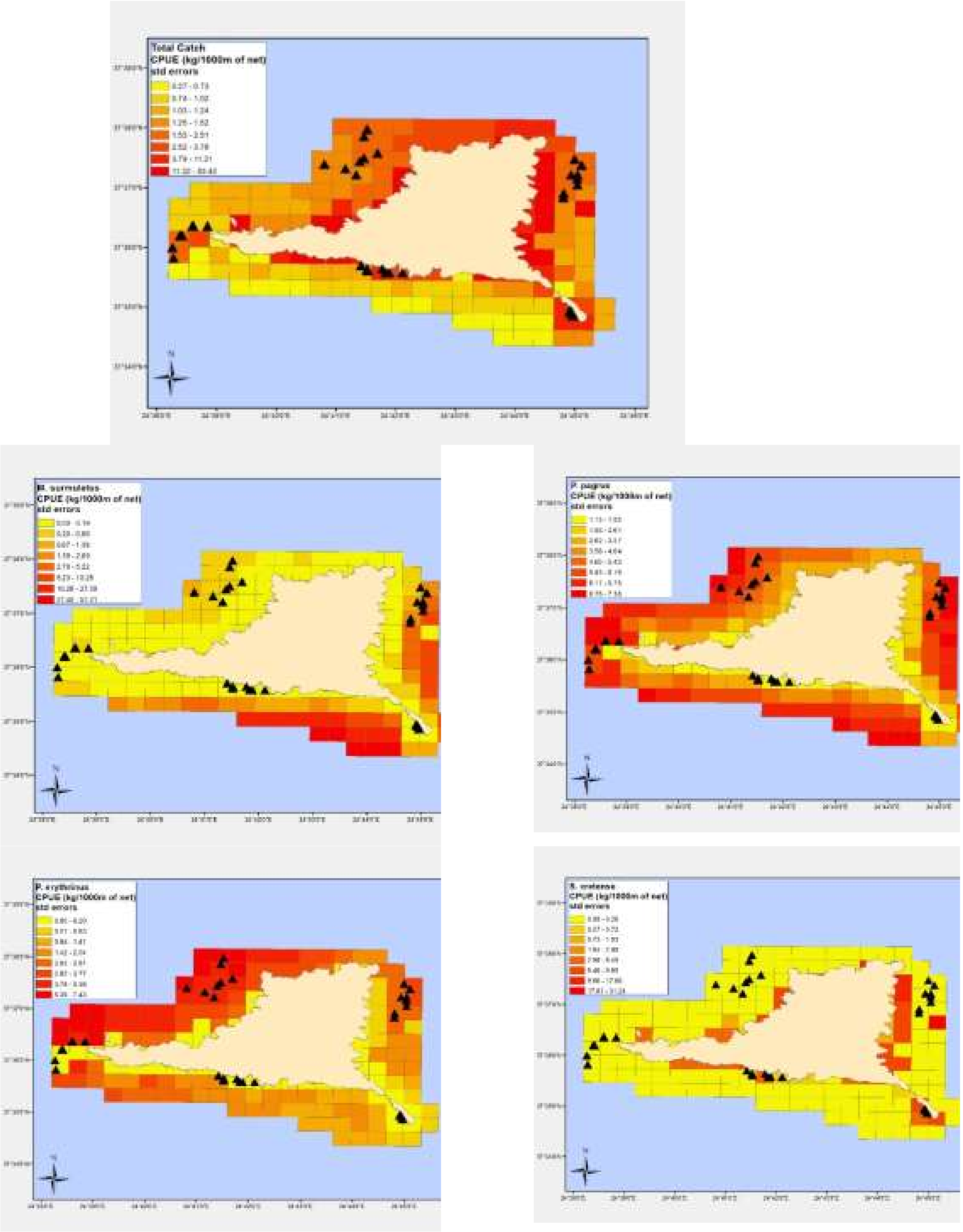

Fig. S1. Spatially predicted standard errors of relative biomass (kg of fish/1000 m of trammel net) for Total catch (top), striped red mullet (mid-left), porgy (mid-right), common Pandora (bottom-left) and Mediterranean parrotfish (bottom-right) around GyarosMPA (sampling locations are depicted in black triangles).

(Greece basemap reprinted from Kavadas et al., 2012 - https://doi.org/10.12681/mms.324 under a CC BY license, with permission from Stefanos Kavadas-HCMR, original copyright 2004)

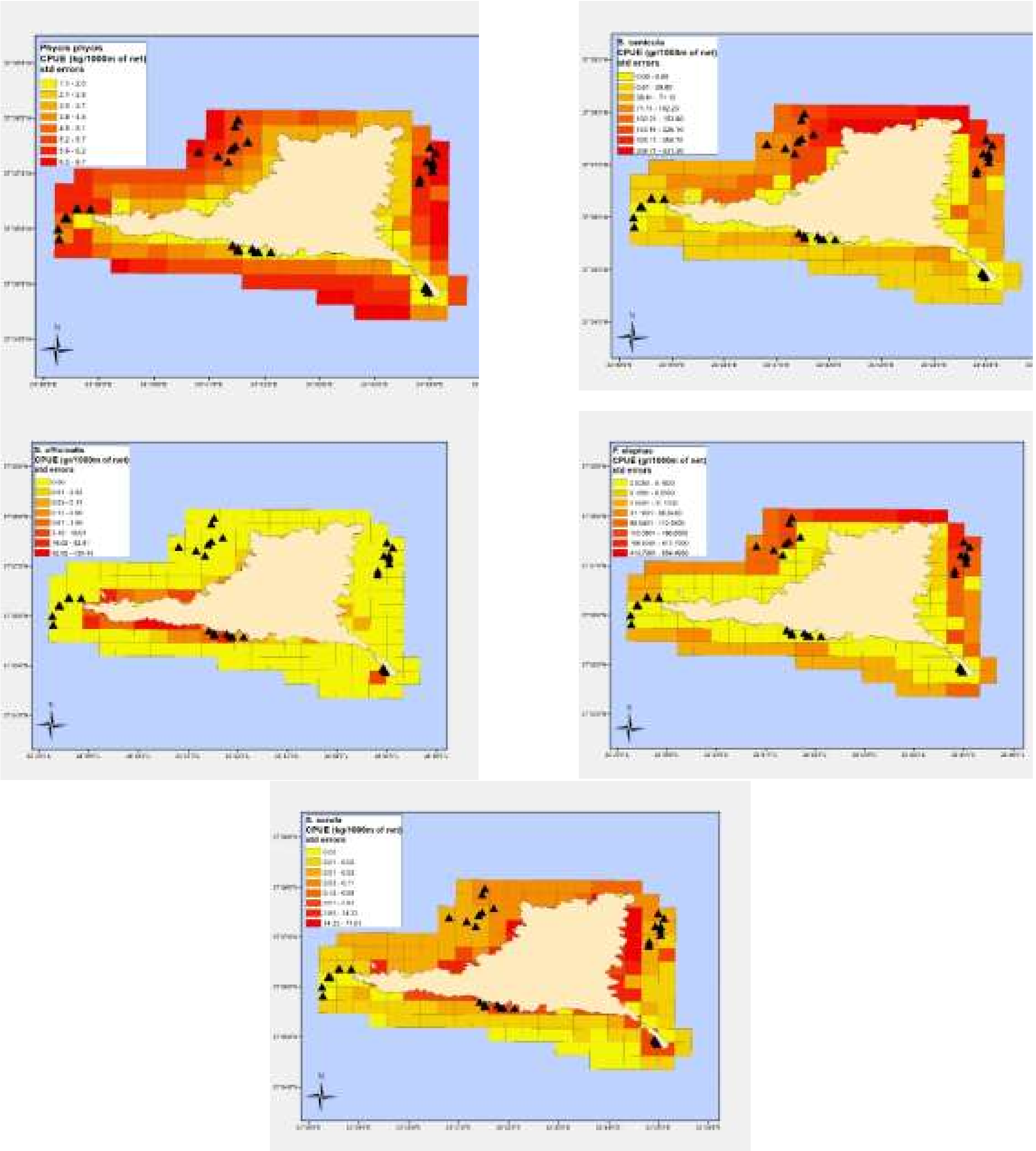

Fig. S2. Spatially predicted standard errors of relative biomass (kg of fish/1000 m of trammel net) for forkbeard (top-left), spotted dogfish (top-right), cuttlefish (mid-left), spiny lobster (mid-right) and red scorpionfish (bottom) around GyarosMPA (sampling locations are depicted in black triangles).

(Greece basemap reprinted from Kavadas et al., 2012 - https://doi.org/10.12681/mms.324 under a CC BY license, with permission from Stefanos Kavadas-HCMR, original copyright 2004)
